# Differential immune gene expression in rainbow trout, *Oncorhynchus mykiss* (walbaum), exposed to five pathogens: *Aeromonas salmonicida, Flavobacterium psychrophilum, Vibrio anguillarum, Yersinia ruckeri and Ichthyophthirius multifiliis*

**DOI:** 10.1016/j.cirep.2024.200166

**Published:** 2024-09-12

**Authors:** Per Walter Kania, Kurt Buchmann

**Affiliations:** Laboratory of Aquatic Pathobiology, Department of Veterinary and Animal Sciences, Faculty of Health and Medical Sciences, University of Copenhagen, Stigbojlen 7, DK-1870 Frederiksberg C, Denmark

**Keywords:** Immune response, Gene expression, Pathogen challenge, Principal component analysis (PCA), Rainbow trout

## Abstract

Rainbow trout, *Oncorhynchus mykiss*, originating from the same outbred population and reared under the same conditions, were bath exposed to five different pathogens (*Aeromonas salmonicida, Flavobacterium psychrophilum, Vibrio anguillarum, Y. ruckeri, Ichthyophthirius multifiliis*). The exposures were conducted as independent experiments with non-exposed fish as time-point controls. At peak of infections, fish showing significant clinical signs, fish without clinical signs, and time-point control fish were euthanized, and gills, spleen, and liver were sampled for gene expression analysis. At the end of the infection course, i.e. a period without any disease signs, surviving fish were sampled as well. Genes encoding 14 cytokines, 5 immune cell markers, 5 immune effector molecules and 3 reference/housekeeping genes were analyzed. The host fish exhibited different reaction patterns dependent on the pathogen. Especially trout infected with *I. multifiliis* showed a characteristic Th2 expression signature in gills, whereas only innate responses occurred in internal organs. Rainbow trout exposed to *F. psychrophilum* displayed a relatively lower expression of immune genes contrasting the host responses to *A. salmonicida, V. anguillarum* and *Y. ruckeri.* The bacterial pathogens induced a relatively similar expression profile in internal organs of trout with a few exceptions. Thus, *F. psychrophilum* induced upregulation of genes earlier in gills than internal organs, and *A. salmonicida* infected fish showed a notable downregulation of immune genes in gills. The differential expression patterns may reflect the different invasion mechanisms and target tissues associated with the five pathogens.

## Highlights

• Immune responses of rainbow trout towards five pathogens were described and compared.
• Infections were associated with specific gene expression profiles.
• The gene expressions clustered as Th1, Th2, Th17 and innate responses.
• Pathogen invasion routes and strategies may explain differences.

## Introduction

Rainbow trout (*Oncorhynchus mykiss*) in aquaculture enterprises suffer from various diseases, which challenge health and welfare of the production fish and cause substantial economic losses at farm level. Some of the most serious bacterial pathogens comprise *Aeromonas salmonicida* causing furunculosis [1], *Flavobacterium psychrophilum*, which is associated with Bacterial Cold Water Disease (BCWD) [2], *Vibrio anguillarum,* the causative agent of vibriosis [3], and *Y. ruckeri,* which is responsible for yersiniosis (Enteric Red Mouth Disease, ERM) [4]. One of the most pathogenic parasites in freshwater trout farming is *Ichthyophthirius multifiliis,* often termed ICH, which is a parasitic ciliate eliciting ichthyophthiriosis or White Spot Disease (WSD) [[5], [6], [7], [8], [9]]. With focus on these five pathogens, we have conducted a series of controlled experimental infections of rainbow trout and measured the expression of immune relevant genes, in gills, spleen, and liver, following pathogen exposure. We sampled fish showing clinical signs, fish without clinical signs and fish surviving the entire course of infection. The fish originated from the same population of outbred rainbow trout from the same farm, and we conducted hatching of fish eggs and rearing of fish under the same environmental conditions in a disease-free hatchery [10]. The experimental conditions, such as infection procedure and temperature, were kept within narrow limits [11,[12], [13], [14], [15]], except for *F. psychrophilum* having a low temperature preference (11 °C) [15,16]. The data from the gene expression analyses were obtained from five previously published studies [11,[12], [13], [14], [15]], all initiated to perform a genome wide association analysis (GWAS) for identification of genomic markers associated with natural resistance. The basic gene expression data were subjected to a Principal Component Analysis (PCA) from where we extracted reaction patterns. Based on the results, we discuss the differential entry routes of the pathogens, activation of different immune pathways, and production of immunomodulatory components.

## Materials and methods

We extracted the qPCR data sets from five previously published studies [11,[12], [13], [14], [15]] under the project TechFish (Innovation Fund Denmark IFD grant no. 8090-00002B). These papers describe in detail all data concerning origin of the fish strains, fish production, infection procedures, sampling, sample preparation, gene expression analysis, and the quantitative real-time method applied.

### Ethics

This study was based on data obtained in previous experiments, and we did not perform new experiments with fish. The data originated from five studies [11,[12], [13], [14], [15]] performed in accordance with the ethical guidelines of the University of Copenhagen, under license 2019-15-0201-01,614 issued from the Experimental Animal Inspectorate, Committee for Experimental Animals, Ministry of Environment and Food, Denmark.

### Fish

The trout eggs (eyed stage) originated from outbred fish at a rainbow trout farm (Aquasearch ova ApS, Jutland, Denmark) and were transferred for hatching and further rearing at the Bornholms Salmon Hatchery (Aqua Baltic, Nexø, Denmark), which is a disease free recirculated fish culture system [10]. When fish reached a size suitable for challenge with a specific pathogen, they were transported (3 h transport in cooled oxygenated freshwater) to the experimental infection facilities at the Laboratory for Aquatic Pathobiology, Frederiksberg Campus, University of Copenhagen, Denmark. Fish were then acclimatized 14 d before exposure to pathogens. A total of 4576 rainbow trout were used for the five challenge studies. Details on the production of pathogens and exposure methods (bath) have been described previously [11,[12], [13], [14], [15]]. The number of fish and their body sizes are shown in Table 1. Fish were fed daily at a rate of 1% (AS, FP, IM) or 1.5% (VA and YR) with pelleted feed (INICIO 917, protein 48%, lipid 23%, carbohydrate 13%) (Biomar, Denmark). Only a part of the involved fish was used for gene expression analyses in the present work. The additional fish were applied for genotyping studies [11,[12], [13], [14], [15]].

**Table 1:** Details on number of fish, fish sizes, age (degree-days) post hatch, temperature at challenge and sampling points. Temperature, size of fish and sampling time points vary due to the nature of the pathogens used. At the 1st sampling when the infection peaked, gill, liver, and spleen were sample from 15 fish with no clinical signs (NCS), 15 fish with clinical signs (CS), and 15 uninfected fish (Ctrl1). At the second sampling when no further mortalities were observed, 15 fish (Survivors) and 15 uninfected fish (Ctrl2) the same organs were sampled. In total 15 × 5 × 3 × 5 = 1125 organs were sampled for qPCR. Due to the slow progress of disease in the case of *F. psychrophilum*, sampling of diseased fish was conducted over 4 days. ^a^ indicates the number of fish exposed to pathogen; unexposed controls were included in all 5 cases but are not included in this table. ^b^ indicates fish age (degree-days) at the start of exposure to pathogens. The term dpe indicates Days Post Exposure. BCWD, ERM and WSD indicate Bacterial Cold-Water Disease, Enteric Red Mouth Disease, and White Spot Disease, respectively. ^c^ Indication of the original studies.

Pathogen	*Aeromonas* *salmonicida*	*Flavobacterium* *psychrophilum*	*Vibrio* *anguillarum*	*Y. ruckeri* O1, biotype 2	*Ichthyophthirius* *multifiliis*
Alias	AS	FP	VA	YR	ICH
Disease	Furunculosis	BCWD	Vibriosis	ERM	WSD
No. of fish ^a^	798	1000	669	1050	1059
Mean weight	8.0 g	3.3 g	12 g	2.5 g	4.6 g
Mean length	8.5 cm	6.9 cm	NA	NA	7.6 cm
Degree-days ^b^	2050	1100	3050	975	1700
Temperature	18 °C	11 °C	18 °C	19 °C	19 °C
1st sampling	3 dpe	11–14 dpe	3 dpe	7 dpe	17 dpe
2nd sampling	13 dpe	40 dpe	11 dpe	20 dpe	21 dpe
Reference ^c^	[14]	[15]	[13]	[11]	[12]

### Challenge and sampling

The five primary studies used the same basic experimental setup, the same water source (municipal water, Frederiksberg, Denmark), and the same infection method (bath). Sampling and processing of samples applied identical reagents and instruments. The experiments differed regarding fish size, pathogen, and temperature at challenge (Table 1). During exposure to pathogens, the fish were surveyed every second hour around the clock throughout the entire course of infection. Recording of clinical signs secured that we could remove moribund fish before they succumbed, a procedure limiting pain and suffering of the experimental animals. Thus, moribund fish with predefined clinical signs [11,[12], [13], [14], [15]] were taken out of the tanks and euthanized using an overdose of MS222 (300 mg/L) (cat.no. A5040, Merck Life Science ApS, Denmark) and recorded as mortality. The first sampling time point was when morbidities rose exponentially. Gills, liver, and spleen from 15 fish showing clinical signs (CS group), 15 fish having no clinical signs (NCS group), and 15 un-exposed control fish (Ctrl1) were sampled. The second sampling occurred when morbidity development reached the plateau phase, and no disease sign were observed. Then gills, liver, and spleen from 15 fish defined as survivors (Surv) and 15 un-exposed control fish (Ctrl2) were sampled. The sampled organs were conserved in RNAlater (cat.no. R0901, Merck Life Science ApS, Denmark) until isolation of RNA, cDNA synthesis and gene expression analysis by real-time quantitative PCR (qPCR) [11,[12], [13], [14], [15]].

### Data analysis

#### Gene expression analysis by real-time quantitative PCR (qPCR)

Quantitative real-time PCR (qPCR) targeting the same 24 immune relevant genes was performed using the same AriaMX real-time PCR machine with the same settings. Based on results from NormFinder [17], an average of the same 3 reference / housekeeping genes was used for normalization. Expression of pathogen specific genes were used to assess the infection level which was calculated as 2^−ΔCq^.

All qPCR assays used annealing temperatures at 60 °C. Primer and probe conditions are listed in the supplementary material file S1 (S1_Primers and Probes). All qPCR assays had efficiencies within 100±5 %, which allowed the use of the simplified 2^−ΔΔCq^ method for estimating relative fold change [18,19]. The ΔCq values used for the present analysis were achieved from the previously published studies [11,[12], [13], [14], [15]]. Four of the 1125 samples gained ≤5 Cq values out of the 24 genes analyzed, and these few values were quite high compared to their replica; all the other samples gained Cq values for at least 12 genes. This indicates that the processing of these four samples might have failed. To avoid any bias from these samples, the four samples were not used for further analyses. None of the samples were from the same group, thus all groups contained at least 14 samples. To ensure uniform data analysis, data were re-analyzed, and new statistical tests included. Fold values are exponential data and do not follow a normal distribution. Therefore, we used the ΔΔCq values for the analyses, as they represent the -log^2^ transformations of folds. Testing for differences of fold change between the experimental groups at sample time point 1 and 2 was performed using one-way ANOVA with Tukey's multiple comparisons test and Students’ *t*-test, respectively (i.e. quantitative assessment); significant gene regulation was only considered when both *p* < 0.05 and fold change was at least 2. In 47 of 1440 (≈3.2 %) tested comparisons at least one group achieved less than 3 Cq values. Therefore, in these cases, the non-parametric test Kruskal-Wallis test with Dunn's multiple comparisons test and the Mann-Whitney test, was applied using the absence / presence of Cq values (i.e. qualitative assessment); here significant gene regulation was considered when *p* < 0.05. The statistical analysis was done using GraphPad Prism 10.0.2. Differences in gene expression between the five pathogens were achieved by calculating ΔΔΔCq (ΔΔCq of pathogen of interest subtracted ΔΔCq of reference pathogen) and then calculating fold change by 2^−ΔΔΔCq^. In this manner, we compared all five pathogens individually to the other four pathogens. Testing for significant difference was done as described above.

#### Principal component analysis (PCA)

A PCA was performed using the -ΔΔCq values of the entire dataset. Folds do not exhibit a Gaussian / normal distribution, whereas the –ΔΔCq representing the log^2^ transformation of the folds do. Three dependent variables were defined as 1) Pathogens with the five values AS (*Aeromonas salmonicida*), FP (*Flavobacterium psychrophilum*), ICH (*Ichthyophthirius multifiliis)*, VA (*V. anguillarum*), and YR (*Y. ruckeri*); 2) Organs with the three values Gill, Liver, and Spleen; 3) Sampling groups with five values NCS, CS, Surv, Ctrl1 (uninfected controls for NSC and CS), and Ctrl2 (uninfected controls for Surv). The 24 immune related genes served as independent variables. PCA was performed using the “Reduction Dimension” tool “Factor” of the software IBM SPSS® Statistics v28.0.0.0 (190). The pattern Matrix was extracted and used for construction of component plot (gene distribution) in GraphPad Prism, and to calculate PC-scores (the individual samples contribution to the component plot) using Excel and GrapPad Prism.

Hierarchical Cluster Analyses were performed using the data of the first three principal components (PC) of the Pattern Matrix obtained using the software IBM SPSS® Statistics v28.0.0.0. The classify / Hierarchical Cluster option was used by setting the number of clusters to 5, the Cluster method to “Centroid Linkage”, and Measure to “Squared Euclidean distance”.

#### Classification trees

Using the same dataset as in PCA (section 2.3.2), classification trees were constructed using the “Classify” tool of the software IBM SPSS® Statistics v28.0.0.0. The genes were used individually as dependent variables and in case of all genes, pathogen, organ, and group were independent variables. We applied the non-parametric growing method CHAID (Chi-squared Automatic Interaction Detection). At each step, CHAID chooses the independent variable (here pathogen, organ, or group) with the strongest interaction with the dependent variable. Branches with *p* > 0.05 were merged.

### Immune relevant genes subjected to expression analysis

We selected twenty-four genes to cover basic, innate and adaptive immune responses. Some of the immune factors encoded by the genes may contribute to different immune pathways, but based on hierarchical Cluster Analysis (see 3.2.1) we divided them into subgroups due to their association with different types of immune responses (Table 2).

**Table 2:** Grouping of genes. In the Principal Component Analysis (PCA), the three Pleiotropic genes did not form a clear and independent cluster (Fig. 2, Fig. 2). In the further analysis, these gene were reallocated into the Innate group (IL-6 and IL-10) and into the th1 group (TGFß) as illustrated by horizontal arrows. Th indicate T-helper like cell response. The Innate response may be divided into two subgroups: pro-inflammatory response (IL-1ß, IL-6, IL8, and IL10) and Acute phase proteins (cathelicidins 1 & 2, Lysozyme, and SAA).

Innate	Pleiotropic	Th1 like	Th2 like	Th17 like
Cathelicidin1	←IL-6	IFNγ	IL-4/13a	C3
Cathelicidin2	←IL-10	IL12	IgDm	IL17A_F2
Lysozyme	TGFß→	TNFα	IgDs	IL17C1
SAA			IgM	Il17C2
IL-1ß			IgT	IL-2
IL-8			TCRß	IL-22

## Results

### Progress of mortalities and pathogen levels

**Fig. 1:**
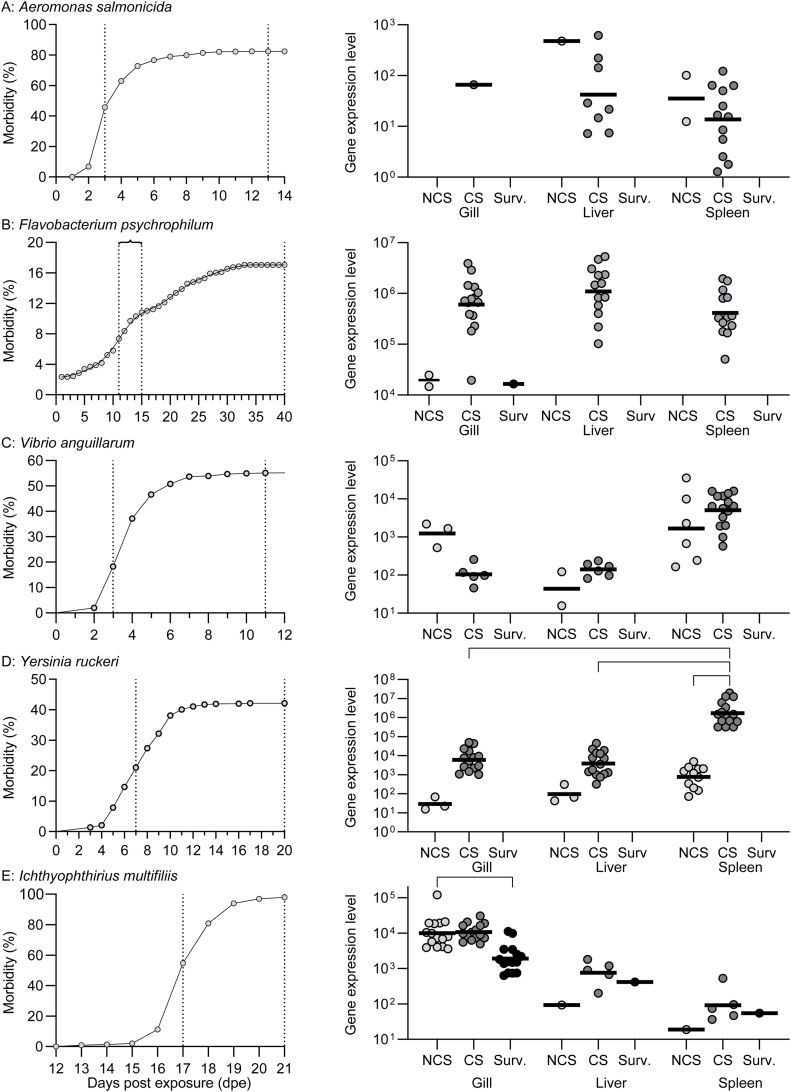
Morbidity curves & Pathogen levels. Left column: In all cases the exposure towards pathogens were preceded by 14 days of acclimatization at experimental temperatures. The vertical dotted lines indicate the two sampling time points (data adopted from [11,[12], [13], [14], [15]]). In the case of the parasite *Flavobacterium* no stressor was applied before exposure, and the progress of disease was quite slow; thus, to get enough samples the first sampling was conducted over 4 days. Right column: Pathogen levels were calculated as2^−Δ^^Cq^ × 10^7^; brackets indicate significant differences (two-way ANOVA with Tukey's multiple comparisons test, *p* < 0.05).

The morbidity curves for rainbow trout exposed to the five pathogens (Fig. 1) exhibit a marked morbidity for all pathogens following exposure. Generally, a lag-phase occurs before disease signs developed and the morbidity peaked. The sampling of fish with clinical signs was conducted during the exponential phase (dashed vertical lines). The bacteria YR, AS and VA propagated very fast and induced disease within a few days (short lag-phase), whereas for the other two pathogens the infection courses were extended, and disease sign peaks were observed after 10–15 dpe (FP) and 17 dpe (ICH) (longer lag-phase). In the case of ICH infection, the remaining fish (2 %) at day 21 were infected but classified as survivors.

Assessment of pathogen levels (Fig. 1 right column) was based on Real-time quantitative PCR using pathogen specific genes (Suppl. Material S1_Primers and Probes) and by calculating 2^-Δ Cq^ x 10^7^. We found significant differences for YR exposed fish with regard to CS compared to NCS of spleen, CS of liver, and CS of gill. Regarding ICH, we merely recorded a significant difference between NCS and Surv in the gills. The three remaining pathogens exhibited no significant differences.

### Expression of genes analyzed by real-time quantitative PCR (qPCR)

The overall immune gene expression study targeted the same 24 immune related genes in all host-pathogen systems studied. However, the sequence, pattern, and organ distribution of the reactions varied according to the pathogen (Table 1).

### Principal component analysis (PCA) using -ΔΔCq values

**Fig. 2:**
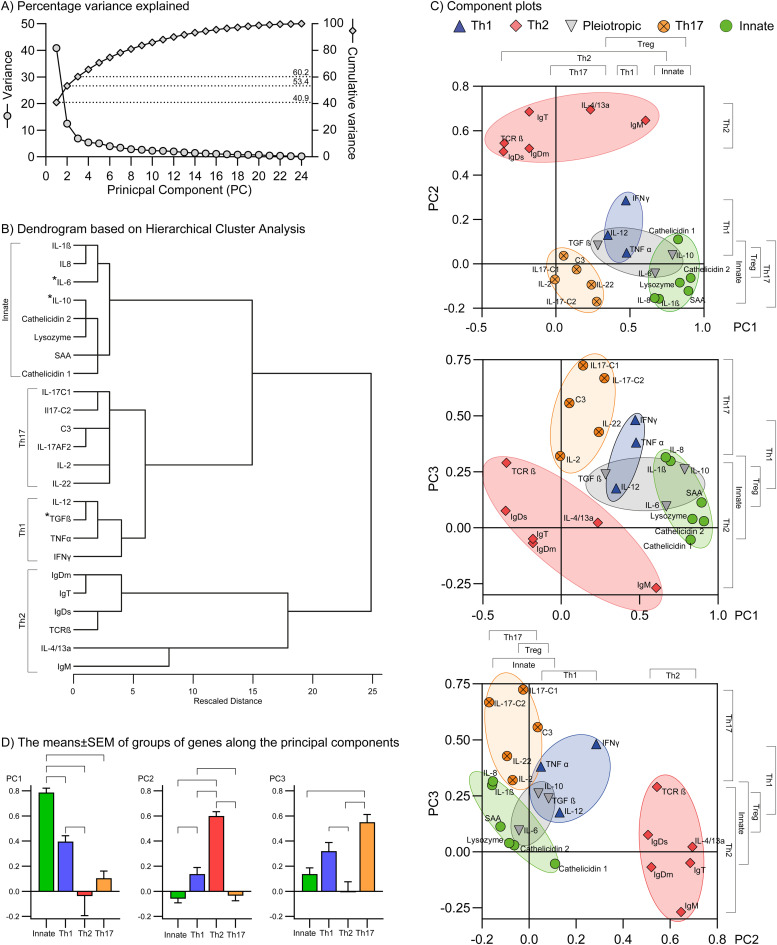
Component plot of the distribution of genes. Based on principal component analysis (PCA), component plot using the first 3 principal components (PC) were constructed. Panel A) The curve of percentage variance flattened after PC3 at which point 60.2 % of the could be explained. Panel B) The cluster analysis was based on the -ΔΔCq values. The three Pleiotropic related genes (marked with asterisks) did not form their own cluster in this cluster analysis but were situated in the Innate cluster (IL-6 and IL-10) and in the Th1 cluster (TGFß). This was also evident in panel C) in which areas indicating the genes of the immune related the pathways Th1, Th2, Pleiotropic, Th17, and Innate response are indicated with blue, red, grey, orange, and green shading, respectively. Panel D) The Pleiotropic related genes were reallocated according to the dendrogram; the brackets indicate significant differences (one-way ANOVA, Šídák's multiple comparisons test, *p* < 0.05) between the groups of genes.

The dataset was adequate for PCA as the Kaiser-Meyer-Olkin Measure of Sampling Adequacy was 0.907 (well above 0.6 and close to the ideal 1) and the p-value of Bartlett's Test of Sphericity was below 0.001, thus < 0.05. The first five PCs had eigenvalues >1 (Kaiser criterion) (Suppl. File S5 (S5_Eigenvalues, Pattern matrix etc.)). However, the curve of a scree plot flattens after PC3, which indicates that the PC4 to PC24 was of minor importance. The first principal component (PC) explained 40.89 % (Fig 2a) of the variance, the second PC 12.49 %, and the third PC 6.86 %; together the first three principal components explained 60.24 % of the differences.

#### Hierarchical cluster analysis

Data from the pattern matrix of the PCA (Suppl. File S5) using the first three PCs was analyzed by Hierarchical Cluster Analysis (Fig. 2b) which revealed to a high degree a pattern of clusters resembling the defined subsets of T helper cells and Innate related genes (Section 2.4, Table 2). The Th2 associated genes were divided into two subclusters, of which IL-4/13a and IgM constituted one subcluster; however, the combined subclusters diverted first in the dendrogram. The Pleiotropic genes were dispersed between the Innate group (IL-6 and IL-10) and the Th1 group (TGF ß) (Fig 2c). Thus, in the further analysis IL-6 and IL-10 were reallocated into the Innate group and TGF ß was reallocated into the Th1 group (Table 2).

#### Component plot of the gene expressions and their association with the immune responses

With data from the pattern matrix of the PCA (Suppl. File S5) using the first three PCs we constructed 2-dimensional component plots showing the distribution of gene expressions (Fig. 2c). The PC4 and PC5 were excluded, as no significant differences between groups of genes were evident (Fig. 2D below in this section). This was supported by the Scree plot (Section 3.2.0 and Suppl. File S5) as the curve flattened after PC3.

PC1. The defined T helper cell groups (section 3.2.1.) exclusive Pleiotropic were to a high degree separated along PC1; from lowest to highest: Th2, Th17, Th1, and Innate. Regarding the Pleiotropic profile, TGF ß aligned with Th1, and IL-6 and IL-10 aligned with Innate. Th2 (exclusive IgM and IL4/13a) and Innate response directed towards low and high values of PC1, respectively.

PC2. Th2 was at the high end. The rest appeared around ±0.2. Again, TGF ß aligned with Th1, IL-6 & IL-10 aligned with Innate. Innate and Th17 directed towards low values of PC2 and Th2 directed toward high values of PC2.

PC3. Th17 directed toward high values of PC3, and IgM of Th2 directed towards low values. To some degree separation along PC3 could be observed, although not as clear as in the case of PC1. From lowest to highest: Th2, Innate, Th1, and Th17. Once more, TGF ß aligned with Th1, whereas IL-6 and IL-10 aligned with the Innate group.

Significant results (*p* < 0.05) of one-way ANOVA with Tukey's multiple comparisons test using the values of the PCs and dividing of the genes into the four groups based on the dendrogram (Fig. 2b) are indicated by brackets on Fig. 2d.

Fig. 2d illustrates the means of the four gene groups, defined by the dendrograms (Fig. 2b), along the first three PCs. Only PC1, 2, and 3 exhibited significant differences (one-way ANOVA with Tukey's multiple comparisons test, *p* < 0,05) whereas PC4 and PC5 had none (not shown). The histograms indicated that the groups of Innate, Th2, and Th17 were the main factors driving the PC1, PC2, and PC3 in positive direction, respectively. The fourth group, Th1 response, was in all three cases the secondary driver, which was reflected by the position in the component plots (Fig. 2c) of the Th1 group between the three other gene groups.

With respect to PC1, the Innate group differed significantly from the three other groups, and also Th1 and Th2 were significantly different from each other. With respect to PC2, the Th2 response differed significantly from the three other groups; besides, the Th1 group differed from the Innate group. With respect to PC3, the Th17 group differed from Innate and Th2; the Th1 group and the Th2 group differed again from each other. The dendrogram and histograms strongly supported the indicated clustering of the component plots (Fig. 2, Fig. 2).

Overall. The four groups (Th1, Th2, Th17, and Innate as defined by the dendrogram, Fig. 2b) formed 4 discrete clusters using 2 dimensional graphs (Fig. 2c). IL-6 and IL-10 of the Pleiotropic group clustered to Innate. TGF ß clustered to Th1 but was also situated in the vicinity of Th17. IL-1ß and IL-8 were on top of each other which is consistent with the role of IL-1ß as an inducer of IL-8 synthesis [20]. In all 3 plots Th2, Th17, and Innate constituted the tips of a triangle with Th1 placed between themes. These observations supported the clustering seen in the dendrogram based on Hierarchical Cluster Analysis (Fig. 2b), like the significant differences obtained when comparing the groups of genes using one-way ANOVA (Fig. 2d).

### Principal components (PC) scores

**Fig. 3:**
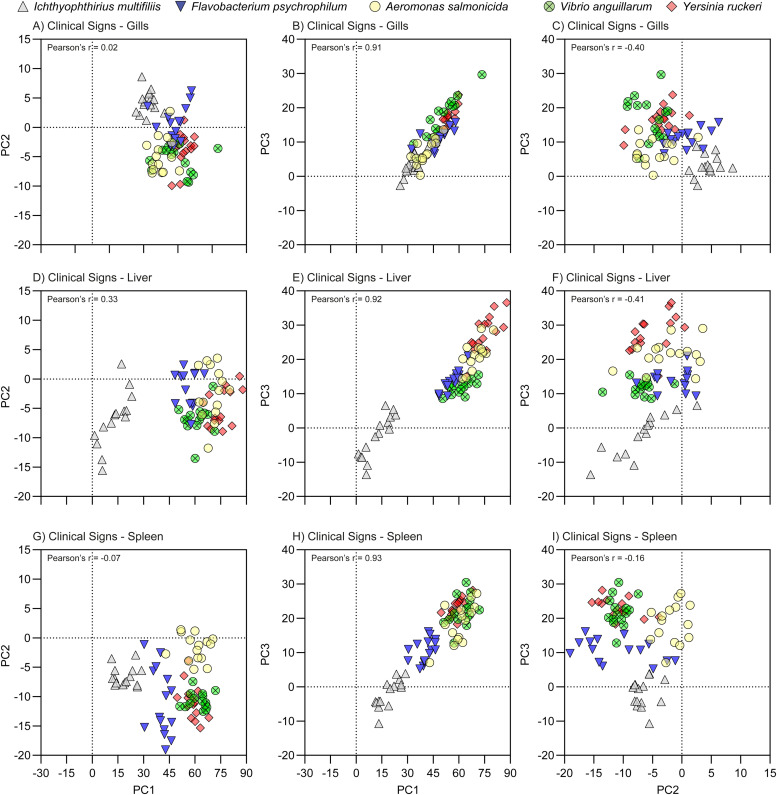
PC score plots of the pathogens, sampling group Clinical Sign. The plots indicate the contributions of the pathogens to the Component Plot (Fig. 2) and are based on PCA performed using the -ΔΔCq values relative to the uninfected controls. The -ΔΔCq value is log^2^ transformed folds, which is exponential data and do not have a Gaussian distribution. See Fig. 6 for significant differences between the pathogens.

**Fig. 4:**
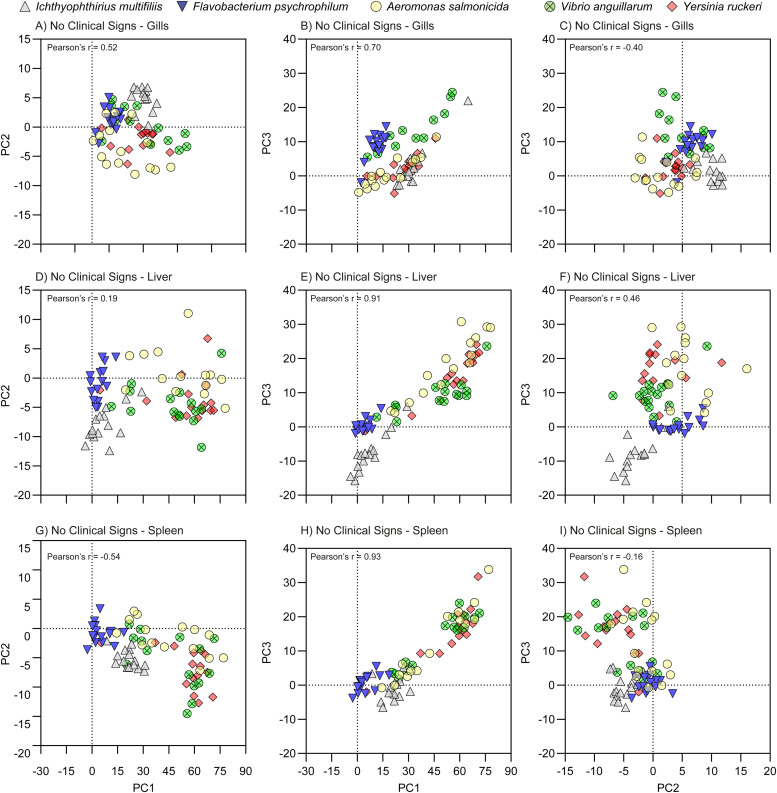
PC score plots of the pathogens, sampling group No Clinical Sign. The plots indicate the contributions of the pathogens to the Component Plot (Fig. 2) and are based on PCA performed using the -ΔΔCq values relative to the uninfected controls. The -ΔΔCq value is log^2^ transformed folds, which is exponential data and do not have a Gaussian distribution.

**Fig. 5:**
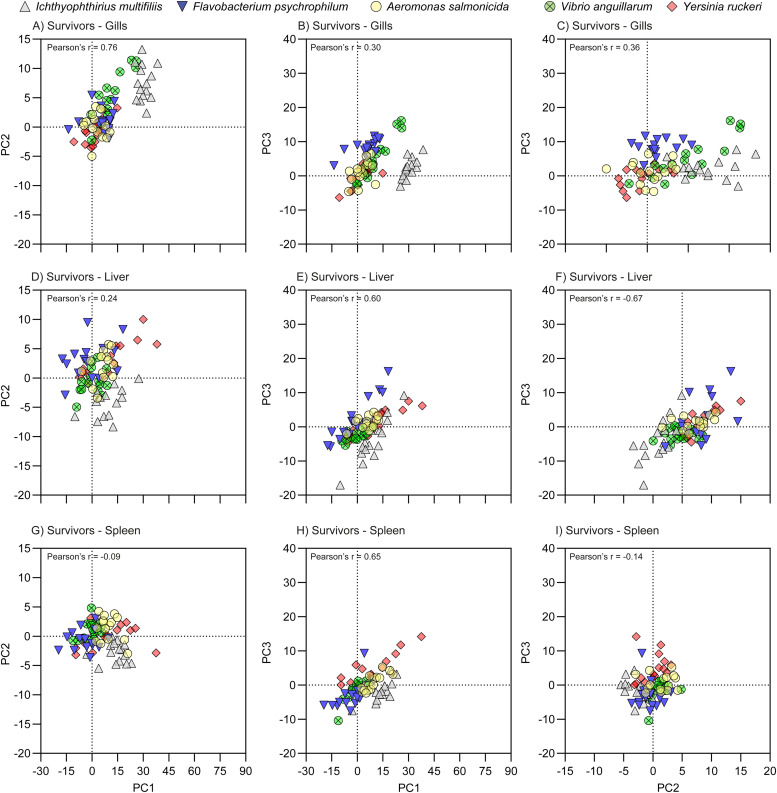
PC score plots of the pathogens, sampling group Survivors. The plots indicate the contributions of the pathogens to the Component Plot (Fig. 2) and are based on PCA performed using the -ΔΔCq values relative to the uninfected controls. The -ΔΔCq value is log^2^ transformed folds, which is exponential data and do not have a Gaussian distribution.

For all samples and each the first three PCs a PC score value was calculated, i.e. 1125 × 3 = 3375 values in total. These values represent the contribution of the individual data points to the distributions of genes along the first three PCs and thereby to the component plots of Fig. 2c. The PC score values were tested for correlation between the three first PCs using Pearson's correlation test (Suppl. File S5) and relevant values are shown on Figs. 3, 4, and 5. When using all samples, a strong positive correlation between PC1 and PC3 (Pearson's *r* = 0.87) was seen, whereas PC1 vs PC2 and PC1 vs PC2 had weak negative correlations (*r*=−0.28 and *r*=−0.10, respectively). In case of PC1 vs PC3, Control groups the overall r was 0.73 with range [0.62;81], NCS overall r was 0.83 with range [0.7;91], CS overall r was 0.91 with range [0.90;93], and finely Surv overall r was 0.53 with range [0.30;65]

Overall, in most cases PC1 and PC3 were strongly positively correlated (*p* > 0.75 in 6 combinations of Groups and Organs) and weakly positive correlated (*p* > 0.05, 2 combinations). In only one case they were not correlated (Gills of Surv, *p* = 0.30). Relevant Pearson's r values are also shown Figs. 3, 4, and 5.

PC score plots were produced using the Pattern Matrix and the -ΔΔCq values to identify factors associated with specific patterns of gene expression, whereby the effects of pathogen type, infection status and organ were visualized.

#### PC score plots focusing on sampling groups

The first approach focused on differences between treatment groups of the individual pathogens (Suppl. File S6 (SF. 6)). Survivors and unexposed controls generally grouped together around the center (0;0), except for parasite (ICH) exposed fish in which CS, NCS and Survivors grouped together around the center. In the fish exposed to bacteria, the CS diverted mostly from the Ctrl and survivors, and NCS generally showed an intermediate expression pattern located between CS and survivors, except in the case of the slow progressing FP, in which the NCS clustered together with Ctrl groups. However, in the PC3 of gills, NCS and survivors of FP grouped together with CS groups, but only slightly above Ctrl group (Suppl. File S6b).

The control groups were omitted in the rest of PC score plots, as the mean of -ΔΔCq in all cases were zero, whereby the average contribution of the control groups to the variation was zero.

#### PC score plots focusing on pathogens

**Fig. 6:**
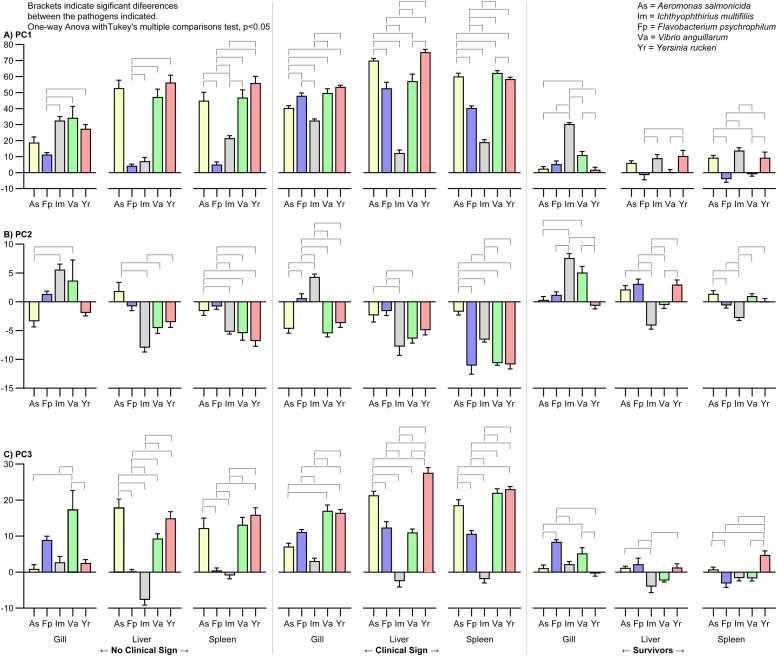
Histograms of the PC score of the pathogens. The brackets indicate significant differences between the pathogens (one-way ANOVA with Tukey's multiple comparisons test and *p* < 0.05). The primary drivers of PC1, PC1, and PC3 were Innate, Th2, and Th17 groups of genes, respectively; the Th2 group was the secondary driver of all three PC's.

The contribution of the pathogen type on the individual sampling groups (Figs. 3, 4, and 5) and on organs (Fig. 6) was evaluated by one-way ANOVA, *p* < 0.05.

The linear trend seen in the plots of PC1 vs PC3 CS (Fig. 3) and NCS (Fig. 4) was in all cases (except for NCS-Gills) significant, as the regression coefficients into power of 2 were above 90 %, and Spearman correlation test resulted in p values <0.0001 for all five pathogens and r values between 0.80 to 0.93.

**Fig. 7:**
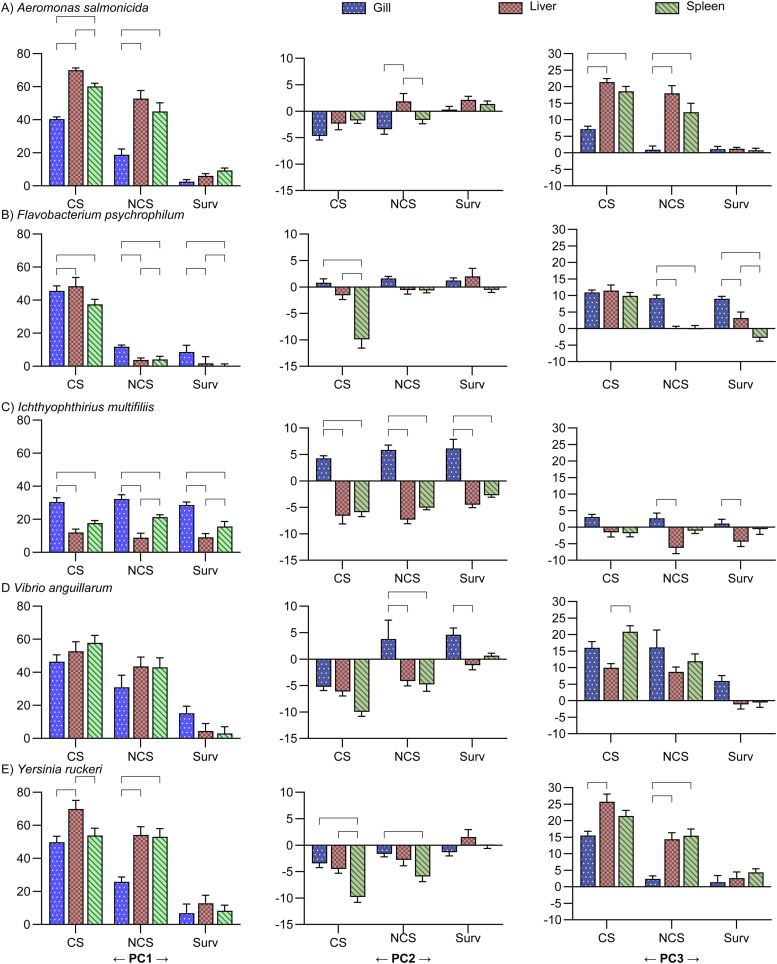
Histograms of the PC score of the organs. The Brackets indicate significant differences between organs (one-way ANOVA with Tukey's multiple comparisons test and *p* < 0.05). Se Suppl. File S7 for two-dimensional PC score plots of the organs. The primary drivers of PC1, PC1, and PC3 were Innate, Th2, and Th17 groups of genes, respectively; the Th2 group was the secondary driver of all three PC's.

The overall contribution of all samples to the PC2 (primary Th2-like) was quite low, range [−8.0;7.6] as compared to PC1 (primary Innate) [−11;75] and PC3 (primary Th17-like) [−7.8;28]. In the NCS, the slowly progressing pathogen FP contributed least to the PC score plots (Fig. 6). The contribution of the NCS, CS, and Surv of IM in gill was significantly higher than in liver and spleen for PC1, PC2, and partly PC3 (Fig. 7c). In case of NCS of FP, even though low, the contribution to PC1 and PC3 was significantly higher in the gills as compared to liver and spleen, which may indicate the gills as one entry point (Fig. 7b). The three remaining bacteria AS, VA, and YR contribute most to the positive direction of PC1 and PC3 and to the negative direction of PC2.

In general, the survivors clustered together with the uninfected controls around the center of the plots (0;0;0)(S6_PC score plots of Sampling Groups.pdf). However, especially in the gills and to minor degree in the liver and spleen the survivors of ICH diverted from the centers with respect to PC1 (Innate) and PC2 (Th2-like). In all three organs, this diversion of Surv of ICH infected fish from the fish infected with bacterial pathogens in the PC score plots was in most cases significant (Fig. 6). ICH infected fish overall contributed mostly to the negative direction of PC3.

Surv of FP infected fish were oppositely located when compared to ICH infected fish the PC1 and PC2 axis. This trend was supported by the general low relative expression levels of the Innate response of FP, whereas Surv of ICH infected fish had relative high expressions levels in the Innate response.

The CS groups were in general the major contributors to the positive directions of PC1 and PC3 and to the negative direction of PC2 (S6_PC score plots of Sampling Groups.pdf). In CS groups of the two bacteria AS and YR, the liver contributed significantly more than gill and spleen. For the FP fish only liver contributed significantly more to the gills. In CS fish infected with VA we found no significant differences.

FP deviated in PC2/PC1 plots for the spleen indicating that this bacterium downregulates expression in spleen. A similar reaction was, to some extent, also noted for VA and AS and YR but not for ICH. The effect of the organ was significant. ICH deviated in all organs (gill, liver, spleen) markedly when compared to the bacterial pathogens (Fig. 3, 4, 5, and 6). FP showed again a significantly lower expression when compared to AS, VA and YR.

### Relative fold change gene expression analysis

Detailed analysis of the relative gene expression of the individual pathogen challenge experiments were presented in the original studies [11,[12], [13], [14], [15]]. To ensure a uniform analysis of the pathogen challenges, the -ΔCq values were adopted from these studies and used for the further calculations and statistical testing. For each of the sampling groups (CS, NCS, and Surv), the relative fold changes between the pathogens are presented in Tables 3, S9,S10, and in detail in Supplementary material file 3. For each of the pathogens, the relative fold changes between the groups are presented in Supplementary material file 2. Folds are shown in the graphs as geometrical means multiplied/divided by geometrical SD (exponential nature of folds).

**Table 3:** An overview of the independent variable Pathogen's interaction in the classification trees. 24 trees (supplementary material S4), one for each gene, were constructed using the non-parametric growing method CHAID; branches were collapsed when *p* ≥ 0.05. ^a^ indicates which branch the secondary separation occurred; for the indicated genes, and organs not indicated in the preceding column. ^b^ here genes for which no separation was evident above the tertiary separation is presented.

Primary separation	Secondary separation:	Under the primary branch ^a^	Tertiary separation and below ^b^
IL 2	TNFα	NCS & CS & Surv & Ctrl	C3
IL 4/13A	IL 17 C2	NCS & CS & Surv	IgT
IL 17 C1	IL 22	NCS & CS & Surv	Lysozyme
	IgDs	NCS & CS & Ctrl	
	Cath 2	NCS & CS	
	IL 6	NCS & CS	
	IL 10	NCS & CS	
	IL 17A/F2	NCS & CS	
	IgDs	NCS & Surv & Ctrl	
	IL 1ß	NCS & Surv & Ctrl	
	IL 8	NCS & Surv & Ctrl	
	SAA	NCS & Surv & Ctrl	
	IgM	NCS & Surv	
	Cath 1	NCS	
	INFg	NCS	
	IL12	CS	
	IgDm	Surv & Ctrl	
	TGF ß	Gill	

#### Comparison of folds between the pathogens (ICH, AS, FP, VA, and YR)

The pathogens were compared to each other's by calculating 2^−ΔΔΔCq^, see section 2.3.1 for details. In this manner the diversity (e.g. age and size) of uninfected control groups was considered. These results are presented in the supplementary file S2 (S2_ qPCR - Comparing Pathogens) and overviewed in Tables S10, S11 and S12. In fish infected with the parasite ICH, the genes were generally downregulated when compared to the fish infected with the four bacteria. However, in some cases ICH had the highest expression levels e.g., the innate response of Surv in all three organs. The genes IL-4/13a, IgM, and IgT of the Th2 response were in general upregulated in the gills as compared to the four bacterial pathogens. In spleen of ICH infected fish, the immune globulins were upregulated as compared to FP, VA, and Yr but downregulated as compared to AS. TCR ß were generally downregulated as compared to the four bacterial infections.

AS infected fish differed from other pathogen systems as the expression in gills of most immune genes were significantly downregulated compared to the other pathogens. In contrast, in the internal organs AS induced a significantly higher expression of the genes – both in CS and NCS and even in survivors. This pattern was to a high degree directly opposite to the situation of ICH infected fish.

VA and YR infected fish displayed a range of similarities – with a notable Th1 expression profile – in gills, liver, and spleen. The main difference between these two pathogens was the absence of a Th17 response in the liver of VA infected fish (CS, NCS and survivors).

#### Comparison of folds between groups related to infection status (CS, NCS, SURV, and controls)

The expression of immune genes in gills, liver, and spleen of fish was subjected to real-time quantitative PCR and analyzed by the relative 2^−ΔΔCq^ method [18]. For each pathogen, folds of CS, NCS, and Survivors relative to uninfected controls and fold of CS relative to NCS are presented in the supplementary file S3 (S3_ qPCR - Comparing Groups) and as schematic form in Table S9. Detailed descriptions on the relative fold change for the five individual pathogens were presented previously [11,[12], [13], [14], [15]].

The Innate immune responses were mainly upregulated, although to lesser extent in Surv than CS and NCS, and in Surv of FP the few significant regulations were downregulations. Excluding ICH, another general trend was that CS was more upregulated than NCS (indicated by # in Table S9). However, in the Th17 responses this trend was absent except for IL-2 and IL-22 in gill in VA and YR. The degree of regulations was overall less in Surv than in CS and NCS. The two pleiotropic genes IL-6 and IL-10 exhibited a quite similar expression pattern. The Innate response of survivors differed between the pathogens (Table S9 and Suppl. File S3). ICH had regulation in all genes except IL-17C2 and the highest number of downregulations in all 4 immune response groups, especially in the liver of all three infections groups (CS, NCS, and Surv). However, in ICH the acute phase proteins cathelicidins 1 & 2, lysozyme, and SAA were all upregulated in the liver in contrast to the pro-inflammatory (IL-1ß, IL-6, IL-8, and IL-10) part of the innate response. YR infected fish exhibited only regulations in the acute phase factors (cathelicidins, lysozyme, and SAA) and none in the interleukins. VA infected fish showed only upregulations in the gills (interleukins and acute phase factors). FP infected fish showed more regulations in the innate response as compared to the other responses. One entry point through the gills was indicated by the profound upregulation of innate responses in the gills of NCS as compared to liver and spleen. In CS, the innate response in all three organs was upregulated to a significantly higher level than in NCS. In the liver of CS and in the gills of Surv a Th17-like response was observed. When comparing fish infected by bacteria, we noted that AS induced most regulations. Acute phase factors of Survivors of AS and YR were upregulated to a higher degree than the other two bacteria FP and VA.

With focus on Th1, Th2, and Th17 responses, the ICH infection differed from the bacterial infections by the high degree of relative downregulation in the internal organs liver and spleen. In contrast, the genes in the gills, specifically targeted by the parasite, were upregulated (Suppl. File S2). Apart from the inflammatory innate response the pathways activated was associated with an adaptive response (Th2) e.g. IL-4/13a, IL-6, IL-10, IgM, and IgT in survivors. In contrast, in ICH infected fish, expression in internal organs (liver and spleen) was mostly downregulated except for genes encoding pleiotropic & innate factors (Table S11 & Suppl. File S2).

In contrast, the three bacterial pathogens YR, VA, and AS mostly elicited gene expression in the internal immune organs (spleen and liver). The pathways induced in the fish by the different pathogens were reflected by the classification trees (overview in Table 3, which are based on Suppl. File S4 (S4_Classification trees).

Fish infected with FP differed from the other bacterial infections. It did not only display a lower progression of the infection, but also showed a lower expression level of most immune genes in internal organs (spleen, liver) as compared to AS, VA and YR, both in CS and NCS fish. The immune genes in gills of FP fish were activated earlier compared to expression in internal organs. The FP survivors showed either no regulation or downregulation of the innate immune genes in all organs compared to the other pathogen/host systems, except that for IL-6 and IL-10 and Innate responses of CS were upregulated.

#### Classification trees

The pathways involving various patterns of cytokine expression in the different host-pathogen systems are illustrated by the branching of the classification trees (Suppl. File S4a (S4a_Classification trees). An overview is provided in (Tables 3). Primary separations of branches were all clean, as they were comprised of either Pathogens (IL-2, IL-4/13a, and IL-17C1), Organs (C3, IL-12 and TGF ß) or Sampling groups (the remaining 18 genes). Further details are described in Suppl. File S4b (S4b_Classification trees Description).

## Discussion

In the five studies [11,[12], [13], [14], [15]] constituting the basis of this meta-study, 24 immune relevant genes were chosen for gene expression analysis of gill, liver, and spleen. Other genes could have been included (e.g. genes encoding transcription factors like T-bet, ROT γ, Fox P3, and GATA3), but the selections represent the major immune pathways involved in rainbow trout responses. Likewise, other organ systems (skin, intestine, thymus, head kidney) could have been included as well. However, previous investigations have illustrated that gene expression in gills, spleen and liver broadly reflect the response of trout to a range of pathogens. We therefore consider this comparative analysis, combining data of the five studies, suitable to provide insight into differential reaction patterns of rainbow trout.

The involvement of innate and adaptive immune elements in host fish responses towards a range of bacterial [4,21,[22], [23], [24], [25], [26], [27]], parasitic [6,8,28,29] and viral [[30], [31], [32], [33], [34]] pathogens has been documented during the latest decades by use of classical immunochemical approaches and molecular tools. Specific identification of immune gene regulation, by use of real-time quantitative PCR, has been a successful tool towards understanding of mechanisms in protective immunity and the immune pathways responsible for the reactions [[34], [35], [36]]. Many of the reactions towards different pathogens activate to some extent quite similar immune elements and a finer differentiation of pathways has not always been clear. In addition, various experimental factors and different experimental laboratory settings may affect the outcome of an infection trial. Thus, the genetic background of the fish used, the experimental setting (e.g. fish tank construction, water source, feed type) including environmental factors (e.g. light, temperature, pH, salinity, water quality) are factors, which are likely to influence the outcome of the challenge. The aim of the present comparative study was therefore to reduce confounding effects and achieve a higher resolution when analyzing these elements in infected fish by applying:

1) rainbow trout all belonging to one outbred strain.
2) use of five different pathogens including one parasite.
3) infection performed in the same facility with the same water quality parameters.
4) samples taken at corresponding time points during the infection course (peak of infection (CS, NCS) and from survivors.
5) samples analyzed by identical methods using identical equipment.

When analyzed by PCA, the expression profiles of rainbow trout differed according to the pathogen used for exposure. In addition, the organ involvement involved differed significantly dependent on the pathogen. The gene expression profiles also clustered in a characteristic way, which to a large extent corresponded to the immune categories, such as Th1, Th2, and Th17, which were originally defined in mammals and later in fish [37,38].

The approach gave a clear picture of differential involvement of organs and immune elements dependent on the pathogen applied. The invasion route and infection strategy of the different pathogens differ to a certain extent, which may explain, at least partly, the differential expression patterns. Thus, the parasitic ciliate invades the fish in both gills and epidermis of the skin, whereas the internal organs remain non-infected. The marked response with adaptive elements involved in gills and a relatively low and mainly innate response in internal organs, as shown in this study, reflects the infection biology of the pathogen. The bacterial pathogens invade the internal fish organs after their penetration of the mucosal barrier, and this may explain that the antibacterial responses exhibit several similarities. However, even among bacteria differences were found. Thus, *F. psychrophilum* induced a markedly different profile reflecting an alternative invasion strategy. Significantly higher contributions of gills by NCS and Surv in PC1 (Innate) and PC3 (Th17) and by CS in PC2 (Th2) were observed. This suggests that the gill apparatus is one entry portal of *F. psychrophilum,* and the transition from gills to internal organs is of longer duration.

The skin and gill parasitic ciliate *I. multifillis* [29] (ICH) elicited, as expected, first an inflammatory response in the target organ (gill) followed by expression of genes encoding internal immune elements of an adaptive response (mainly Th2) (Table S11). This complies with previous studies documenting that antibodies produced in gills contribute to protection against this parasite [7,12,29]. The minimal involvement of internal organs (spleen, liver) was noteworthy, which suggests that the protective response mainly is exerted by elements of the mucosal immunity. Thus, previous studies have indicated that rainbow trout is able to raise a protective immune response against ICH [8,29], and involve adaptive processes in central immune organs of the fish. We confirmed that internal organs were indeed involved, but primarily reflected by the elevated expression of the pro-inflammatory part of the innate immune genes (IL-1β, IL-6, IL-8, and IFNγ) in liver and spleen of ICH infected fish and survivors. This suggests that adaptive responses may be established locally in the mucosal immune system (MALT) of gills, but also that sustained protection to some extent may be independent of the internal organs, i.e. liver and spleen, in fish.

The ICH infection elicited markedly different reactions compared to the bacterial infections. This complies with the notion that ICH targets the epidermal sites by penetration and does not invade internal organs. In contrast, the four bacterial pathogens all invade the internal organs, which led to significant regulation in liver and spleen. However, also within this group, comprising four bacterial species, the reactions induced differed, and especially *Flavobacterium psychrophilum* (FP) deviated by eliciting lower levels of immune genes in the internal organs when compared to other bacterial pathogens Tables S10, S11 and S12 (Suppl. File S2_ qPCR - Comparing Pathogens). Responses in fish hosts against this pathogen were studied previously, but the present study places the gene expression in a broader comparative perspective in relation to other bacterial pathogens [23]. The FP bacterium is psychrophilic, and the infection occurs at low temperatures (in this case 11–12 °C). Hence the infection course is also extended when compared to VA, YR and AS infections. Although lowering of the temperature in itself will moderate immune gene expression in rainbow trout [39], the study indicated an effect of FP. The downregulation was seen in relation to un-infected time-point control fish, kept at identical conditions, which suggest that the bacterium in itself has immune-suppressing properties. The upregulation of genes in gills before internal organs may further reflect a more extended invasion process in fish surfaces. Differential response characteristics between bacterial pathogens were indeed shown. This was clear from the fish reactions to YR, which is known to induce a strong up-regulation of a range of immune genes in rainbow trout [40,41] especially in the internal organs. The uptake of the pathogen via the external surfaces may be fast and merely elicit a transient response in gills and skin [40,41], whereas the main processing occurs in the internal organs.

A partly corresponding invasion route is displayed by AS. The trout exposed to AS, when compared to VA and YR fish, showed a general downregulation in gills but a stronger response in liver (complying with a mixed Th1/Th2/Th17response). This could indicate that the pathogens pass easily through the external mucosal barrier, possibly by downgrading the host response at the entry point, whereafter they target primarily the internal organs.

VA induced in liver a downregulation of genes associated with the Th17 pathway in all exposed trout (CS, NCS, survivors) and downregulated the Th2 associated genes in gill, liver, and spleen in CS fish. The differential involvement of organs could be interpreted, not only as a VA pathogen preference for these targets, but also as a specific molecular strategy of the pathogen to circumvent the host immune response [3].

Previous studies have indicated that a certain pathogen may induce a combination of pathways [[14], [15], [16]]. However, it should be noted that various factors, including cytokines, take part in different pathways. Although often assigned as T helper cell (Th) response genes, e.g. IL-12 and IL-23, are also produced by macrophages and dendritic cells, subsequently guiding a T cell response [20]. In addition, in the mammalian immune system, C3 mediates Th1 / Th17 polarization in T-cell activation with IL-2 involvement [42]. It is also noted that some of the genes have a pleiotropic nature. One of the activities of TGF ß is to be a major controller of T-cell differentiation (reviewed in [[43], [44], [45]]). Thus, by performing the hierarchical cluster analysis (Fig. 2b) and component plots (Fig. 2c) this study demonstrated that IL-6 and IL-10 clustered together with the Innate group and, TGF ß clustered together with Th1 group. This was the reason that these pleiotropic genes were allocated to the gene groups Innate (IL-6 and IL-10) and Th1 (TGF ß). The positive correlation seen in the plots of PC1 vs PC3 CS (Fig. 3) and NCS (Fig. 4) may indicate that the Innate response (primary of PC1), the Th17-like (primary of PC3), and to a lesser degree Th1 (secondary of both PC1 and PC3) responses are expressed in an orchestrated manner.

## Conclusions

We experimentally exposed rainbow trout to five different pathogens, one parasite and four bacteria, in separate trials. We reduced confounding effects by using fish of the same genetic origin, the same hatching and rearing conditions, the same infection facilities, identical sampling, and identical gene expression analyses. The comparative study revealed different gene expression profiles in fish dependent on the pathogen. Especially the parasitic ciliate *I. multifiliis*, targeting the host skin and gills, elicited a characteristic response in the gills including adaptive elements, and merely innate reactions in the internal organs. Bacterial pathogens induced to some extent comparable reactions, but *F. psychrophilum* elicited reactions in gills before internal organs, and *A. salmonicida* induced a marked down-regulation of the response in gills. The main reasons for the differential response observed may be 1) the invasion route taken by the pathogen, 2) the target organs in the fish selected by the different pathogens following systemic infection, and 3) specific immune regulating mechanisms applied by the different pathogens. The differential responses in rainbow trout organs in relation to different pathogens suggest that the five different pathogens interact differently with the host.

## CRediT authorship contribution statement

**Per Walter Kania:** Writing – original draft, Visualization, Validation, Methodology, Investigation, Formal analysis, Data curation, Conceptualization. **Kurt Buchmann:** Writing – review & editing, Validation, Resources, Project administration, Funding acquisition, Data curation.

## Declaration of competing interest

The authors declare that they have no known competing financial interests or personal relationships that could have appeared to influence the work reported in this paper.

## Data Availability

Data will be made available on request.

## Appendix Supplementary materials

**Supplementary material file 1.** Primers and probes. All qPCR assays have annealing temperature at 60 °C and efficiencies at 100 %±5 %. Image, application 1

**Supplementary material file 2.** The qPCR results of comparing pathogens to each other is presented in spreadsheet tables. Image, application 2

**Supplementary material file 3.** The qPCR results of comparing sampling groups (CS, NCS, Surv, and controls) to each other is presented in spreadsheet tables. Image, application 3

Supplementary material file 4a. Classification trees. Image, application 4

**Supplementary material file 4b.** Classification trees, description Image, application 5

**Supplementary material file 5.** Eigenvalues, Pattern matrix etc. of the Principal Component Analysis (PCA). Image, application 6

**Supplementary material file 6.** Pc score plots showing the contribution of the sampling groups to the Component plot Fig. 2c. Image, application 7

**Supplementary material file 7.** Pc score plots showing the contribution of the organs to the Component plot Fig. 2c. Image, application 8

Image, application 9

Image, application 10

Image, application 11

Image, application 12
