# Supplementary material for: Differential immune gene expression in rainbow trout, Oncorhynchus mykiss (walbaum), exposed to five pathogens: Aeromonas salmonicida, Flavobacterium psychrophilum, Vibrio anguillarum, Yersinia ruckeri and Ichthyophthirius multifiliis
Source: Comp Immunol Rep. 2024 Sep 12;7:200166. doi: 10.1016/j.cirep.2024.200166 (PMC11437762; doi:10.1016/j.cirep.2024.200166)
Supplement: Supplementary file 1 — Supplementary material file 1. Primers and probes. All qPCR assays have annealing temperature at 60 °C and efficiencies at 100 %±5 %. [file mmc1.docx]

**Table 1**

Primers and probes used for qPCR assays. All nucleotides are from 5’ end (labeled with FAM) to 3’ end (labeled with BHQ1). All the qPCR assays were optimized to have annealing temperature of 60°C and having efficiencies of 100% ± 5%. ^R^ indicates reference genes (housekeepers). ^MS^ indicates the qPCR assay targets both membrane bound and secreted forms. ^1^ The α chain of IL-12 is common to the two isoforms of IL-12.

| **Gene**  **GenBank acc.no.** | **Length**  **Bp** | **Primers**  **5’end to 3’end** | **Probes**  **5’end to 3’end** | **References** |
| --- | --- | --- | --- | --- |
| ^R^ ARP  AY505012 | 106 | Fwd: GAAAATCATCCAATTGCTGGATG  Rev: CTTCCCACGCAAGGACAGA | CTATCCCAAATGTTTCATTGTCGGCGC | [1] |
| ^R^ β-actin  AB196465 | 241 | Fwd: ACATCAAGGAGAAGCTGTGCTAC  Rev: TACGGATGTCCACGTCACAC | CCTCTCTGGAGAAGAGCTACGAGCTG | [2]  Probe [3] |
| ^R^ ELF-1α  [AF498320](http://www.ncbi.nlm.nih.gov/entrez/viewer.fcgi?db=nucleotide&val=20269865) | 63 | Fwd: ACCCTCCTCTTGGTCGTTTC  Rev: TGATGACACCAACAGCAACA | GCTGTGCGTGACATGAGGCA | [4] |
| C3.3 & C3.4  AF271080 / U61753 | 85 | Fwd: ATTGGCCTGTCCAAAACACA  Rev: AGCTTCAGATCAAGGAAGAAGTTC | TGGAATCTGTGTGTCTGAACCCC | [5] |
| Cathelicidin 1A  AY382478 | 189 | Fwd: TCTCTCGTCCTGGGGTT  Rev: GTTGTAGCGTGCTGATCTATG | TAATTGGTCGTCCTGGGGGTGG | [3] |
| Cathelicidin 2A  AY360356 | 135 | Fwd: AAAGATTCCAAGGGGGGT  Rev: CAAAGGGTGTGTTGTGCTGT | GCTCTCGTCCTGGGTTTGGCTCC | [6] |
| IFN γ1 and IFN γ2  FJ184374 / FJ184375 | 68 | Fwd: AAGGGCTGTGATGTGTTTCTG  Rev: TGTACTGAGCGGCATTACTCC | TTGATGGGCTGGATGACTTTAGGA | [7] |
| IgDm  AY870262 | 304 | Fwd: CAGGAGGAAAGTTCGGCATCA  Rev: CCTCAAGGAGCTCTGGTTTGGA | CCACACCACACAGACTCTGGCCCTGAA | [8] |
| IgDs  JQ003979 | 304 | Fwd: TGGCACGCCAGGATTTGAC  Rev: TCAGAATTGAGTGAACGGACAGACA | CCACACCACACAGACTCTGGCCCTGAA | [8] |
| ^MS^ IgM  S63348 / AH014877 | 72 | Fwd: ACCCTCCTCTTGGTCGTTTC  Rev: TGATGACACCAACAGCAACA | TGATGACACCAACAGCAACA | [7] |
| ^MS^ IgT  AY870265 / AY870263 | 73 | Fwd: AGCACCAGGGTGAAACCA  Rev: GCGGTGGGTTCAGAGTCA | AGCAAGACGACCTCCAAAACAGAAC | [7] |
| IL-1β  AJ223954 | 91 | Fwd: ACATTGCCAACCTCATCATCG  Rev: TTGAGCAGGTCCTTGTCCTTG | CATGGAGAGGTTAAAGGGTGGC | [7] |
| IL-2a  FJ571513 | 110 | Fwd: ATGCAACACCACATCAGCAT  Rev: TGCCACGGCCCTACAAAAGA  RE  TGCCACGGCCCTACAAAAGA | TGCCACGGCCCTACAAAAGA | [3] |
| IL-4/13a  AB574337 | 138 | Fwd: ATCCTTCTCCTCTCTGTTGC  Rev: GAGTGTGTGTGTATTGTCCTG | CGCACCGGCAGCATAGAAGT | [9] |
| IL-6a  DQ866150 | 91 | Fwd: ACTCCCCTCTGTCACACACC  Rev: GGCAGACAGGTCCTCCACTA | CCACTGTGCTGATAGGGCTGG | [10] |
| IL-8 isoforms a, b , c, d & e  AY160982 to AY160986 | 69 | Fwd: AGAATGTCAGCCAGCCTTGT  Rev: TCTCAGACTCATCCCCTCAGT | TTGTGCTCCTGGCCCTCCTGA | [10] |
| IL-10a  [AB118099](http://www.ncbi.nlm.nih.gov/entrez/viewer.fcgi?db=nucleotide&val=47678892) | 70 | Fwd: CGACTTTAAATCTCCCATCGAC  Rev: GCATTGGACGATCTCTTTCTTC | CATCGGAAACATCTTCCACGAGCT | [7] |
| ^1^ IL-12 α chain  HE798148 | 84 | Fwd: CAACGGAACACCACATTCAG  Rev: AGCCTGTAGTGAGGCAGCAT | TGCGTGTCTGAGGAACATCCG | [11] |
| IL-17A/F2a  AJ580842 | 158 | Fwd: TCAAAAGCAACGTGTCGAAG  Rev: TCCCTCTGATTCCTCTGTGG | TATGCTGCTGGGCCTGACCA | [11] |
| IL-17c1  CAW30792 | 138 | Fwd: CTGGCGGTACAGCATCGATA  Rev: GAGTTATATCCATAATCTTCGTATTCGGC | CGTGATGTCCGTGCCCTTTGACGATG | [9] |
| IL-17c2  CAW30793 | 134 | Fwd: CTGGCGGTACAGCATCGATA  Rev: CAGAGTTATATGCATGATGTTGGGC | CGTGGTGTCCAGGCCCTTTAATGATG | [9] |
| IL-22  AM748537 | 64 | Fwd: ATGACCACCACCACAGCATT  Rev: ATTCCTTTCCCCTCCTCCAT | CTTTCCGCAAGAAGTTGTCCGAG | [12] |
| Lysozyme  X59491 | 188 | Fwd: GAAACAGCCTGCCCAACT  Rev: GTCCAACACCACACGCTT | ATACCCAGGCCACCAACCGCAACAC | [13] |
| SAA  AM422446 | 79 | Fwd: GGGAGATGATTCAGGGTTCCA  Rev: TTACGTCCCCAGTGGTTAGC | TCGAGGACACGAGGACTCAGCA | [14] |
| TCR-β  AF329700 | 73 | Fwd: TCACCAGCAGACTGAGAGTCC  Rev: AAGCTGACAATGCAGGTGAATC | CCAATGAATGGCACAAACCAGAGAA | [7] |
| TGF-β1a  [X99303](http://www.ncbi.nlm.nih.gov/entrez/viewer.fcgi?db=nucleotide&val=1478246) | 75 | Fwd: TCTGAATGAGTGGCTGCAAG  Rev: GGTTTCCCACAATCACAAGG | CTGGAGAGGAGCAGGGATTCCAAT | [7] |
| TNF-α1 & TNF-α2  AJ277604 / AJ401377 | 75 | Fwd: GGGGACAAACTGTGGACTGA  Rev: GAAGTTCTTGCCCTGCTCTG | GACCAATCGACTGACCGACGTGGA | [10] |
| *aop0* (*A salmonicida*)  DQ386862 | 248 | Fwd: AGCTCATCCAATGTTCGGTATT  Rev: AAGTTCATCG TGCTGTTCCA | GGCGCTCAATCCGGTTTACCCACGG | [15] |
| IAG52A (*I. multifiliis.*)  AF324424 | 240 | Fwd: TTGGAACTGAAACTAACACAGCC  Rev: CTCCACCTGCAATTGCGGTA | TGCTGCTGCTTTCGTTCCTGGTGC | [16] |
| 16S (*F. psycrophilum*))  LC370212 | 248 | Fwd: GTTGCCAGCGAGTCATGT  Rev: AGCACGTGTGTAGCCCAA | AACTGTGAGGAAGGTGGGGATGACG | [17] |
| *recA* (*V. -α*))  LC370212 | 248 | Fwd: ATCGCGGCTCCCTTTAAACA  Rev: AGAGAATCCAGCCGCCGCCATGG | AACTCGGCTGGATTGAGCAG | [18] |
| glnA (Yersinia ruckeri)  AY333067 | 109 | Fwd: TCCAGCACCAAATACGAAGG  Rev: ACATGGCAGAACGCAGATC | AAGGCGGTTACTTCCCGGTTCCC | [19] |

[1] M.K. Purcell, G. Kurath, K.A. Garver, R.P. Herwig, J.R. Winton, Quantitative expression profiling of immune response genes in rainbow trout following infectious haematopoietic necrosis virus (IHNV) infection or DNA vaccination, Fish & shellfish immunology 17(5) (2004) 447-462.

[2] S.H. Sugiura, K. Kelsey, R.P. Ferraris, Molecular and conventional responses of large rainbow trout to dietary phosphorus restriction, Journal of comparative physiology. B, Biochemical, systemic, and environmental physiology 177(4) (2007) 461-72.

[3] S. Zuo, A.M. Karami, J. Ødegård, H. Mathiessen, M.H. Marana, R.M. Jaafar, L. von Gersdorff Jørgensen, M. Abdu, P.W. Kania, I. Dalsgaard, T. Nielsen, K. Buchmann, Immune gene expression and genome-wide association analysis in rainbow trout with different resistance to Yersinia ruckeri infection, Fish & shellfish immunology 106 (2020) 441-450.

[4] H.-C. Ingerslev, E.F. Pettersen, R.A. Jakobsen, C.B. Petersen, H.I. Wergeland, Expression profiling and validation of reference gene candidates in immune relevant tissues and cells from Atlantic salmon (Salmo salar L.), Molecular Immunology 43(8) (2006) 1194-1201.

[5] M.K. Raida, K. Buchmann, Innate immune response in rainbow trout (*Oncorhynchus mykiss*) against primary and secondary infections with *Yersinia ruckeri* O1, Developmental and comparative immunology 33(1) (2009) 35-45.

[6] J. Xueqin, P.W. Kania, K. Buchmann, Comparative effects of four feed types on white spot disease susceptibility and skin immune parameters in rainbow trout, *Oncorhynchus mykiss* (Walbaum), Journal of Fish Diseases 35(2) (2012) 127-135.

[7] M.K. Raida, K. Buchmann, Temperature-dependent expression of immune-relevant genes in rainbow trout following *Yersinia ruckeri* vaccination, Diseases of Aquatic Organisms 77(1) (2007) 41-52.

[8] J. Skov, J.K. Chettri, R.M. Jaafar, P.W. Kania, I. Dalsgaard, K. Buchmann, Effects of soluble immunostimulants on mucosal immune responses in rainbow trout immersion-vaccinated against Yersinia ruckeri, Aquaculture 492 (2018) 237-246.

[9] J.K. Chettri, J.A. Kuhn, R.M. Jaafar, P.W. Kania, O.S. Moller, K. Buchmann, Epidermal response of rainbow trout to Ichthyobodo necator: immunohistochemical and gene expression studies indicate a Th1-/Th2-like switch, J Fish Dis 37(9) (2014) 771-83.

[10] M.K. Raida, K. Buchmann, Bath vaccination of rainbow trout (*Oncorhynchus mykiss* Walbaum) against *Yersinia ruckeri*: effects of temperature on protection and gene expression, Vaccine 26(8) (2008) 1050-62.

[11] R.M. Jaafar, J.K. Chettri, I. Dalsgaard, A. Al-Jubury, P.W. Kania, J. Skov, K. Buchmann, Effects of adjuvant Montanide™ ISA 763 A VG in rainbow trout injection vaccinated against Yersinia ruckeri, Fish & Shellfish Immunology 47(2) (2015) 797-806.

[12] M.M. Olsen, P.W. Kania, R.D. Heinecke, K. Skjoedt, K.J. Rasmussen, K. Buchmann, Cellular and humoral factors involved in the response of rainbow trout gills to *Ichthyophthirius multifiliis* infections: Molecular and immunohistochemical studies, Fish & Shellfish Immunology 30(3) (2011) 859-869.

[13] J.K. Chettri, M.K. Raida, P.W. Kania, K. Buchmann, Differential immune response of rainbow trout (Oncorhynchus mykiss) at early developmental stages (larvae and fry) against the bacterial pathogen Yersinia ruckeri, Developmental & Comparative Immunology 36(2) (2012) 463-474.

[14] J. Skov, P.W. Kania, L. Holten-Andersen, B. Fouz, K. Buchmann, Immunomodulatory effects of dietary beta-1,3-glucan from Euglena gracilis in rainbow trout (Oncorhynchus mykiss) immersion vaccinated against Yersinia ruckeri, Fish & shellfish immunology 33(1) (2012) 111-20.

[15] C. Fernandez-Alvarez, S.F. Gonzalez, Y. Santos, Development of a SYBR green I real-time PCR assay for specific identification of the fish pathogen Aeromonas salmonicida subspecies salmonicida, Applied microbiology and biotechnology 100(24) (2016) 10585-10595.

[16] R. Jaafar, J. Ødegård, H. Mathiessen, A.M. Karami, M.H. Marana, L. von Gersdorff Jørgensen, S. Zuo, T. Nielsen, P.W. Kania, K. Buchmann, Quantitative trait loci (QTL) associated with resistance of rainbow trout Oncorhynchus mykiss against the parasitic ciliate Ichthyophthirius multifiliis, Journal of fish diseases 43(12) (2020) 1591-1602.

[17] H. Mathiessen, Y. Duan, M.H. Marana, S. Zuo, A.M. Karami, R. Jafaar, L. von Gersdorff Jørgensen, P.W. Kania, I. Dalsgaard, L. Madsen, T. Nielsen, F. Grammes, J. Ødegård, V. Macchia, K. Buchmann, Validation of a QTL for Flavobacterium psychrophilum resistance in rainbow trout Oncorhynchus mykiss, Aquaculture Reports 30 (2023) 101573.

[18] A.M. Karami, J. Ødegård, M.H. Marana, S. Zuo, R. Jaafar, H. Mathiessen, L. von Gersdorff Jørgensen, P.W. Kania, I. Dalsgaard, T. Nielsen, K. Buchmann, A Major QTL for Resistance to Vibrio anguillarum in Rainbow Trout, Frontiers in Genetics 11(1731) (2020).

[19] S.E. Keeling, C. Johnston, R. Wallis, C.L. Brosnahan, N. Gudkovs, W.L. McDonald, Development and validation of real-time PCR for the detection of Yersinia ruckeri, Journal of fish diseases 35(2) (2012) 119-25.
