# Supplementary figures and images for: Differential immune gene expression in rainbow trout, Oncorhynchus mykiss (walbaum), exposed to five pathogens: Aeromonas salmonicida, Flavobacterium psychrophilum, Vibrio anguillarum, Yersinia ruckeri and Ichthyophthirius multifiliis
Source: Comp Immunol Rep. 2024 Sep 12;7:200166. doi: 10.1016/j.cirep.2024.200166 (PMC11437762; doi:10.1016/j.cirep.2024.200166)

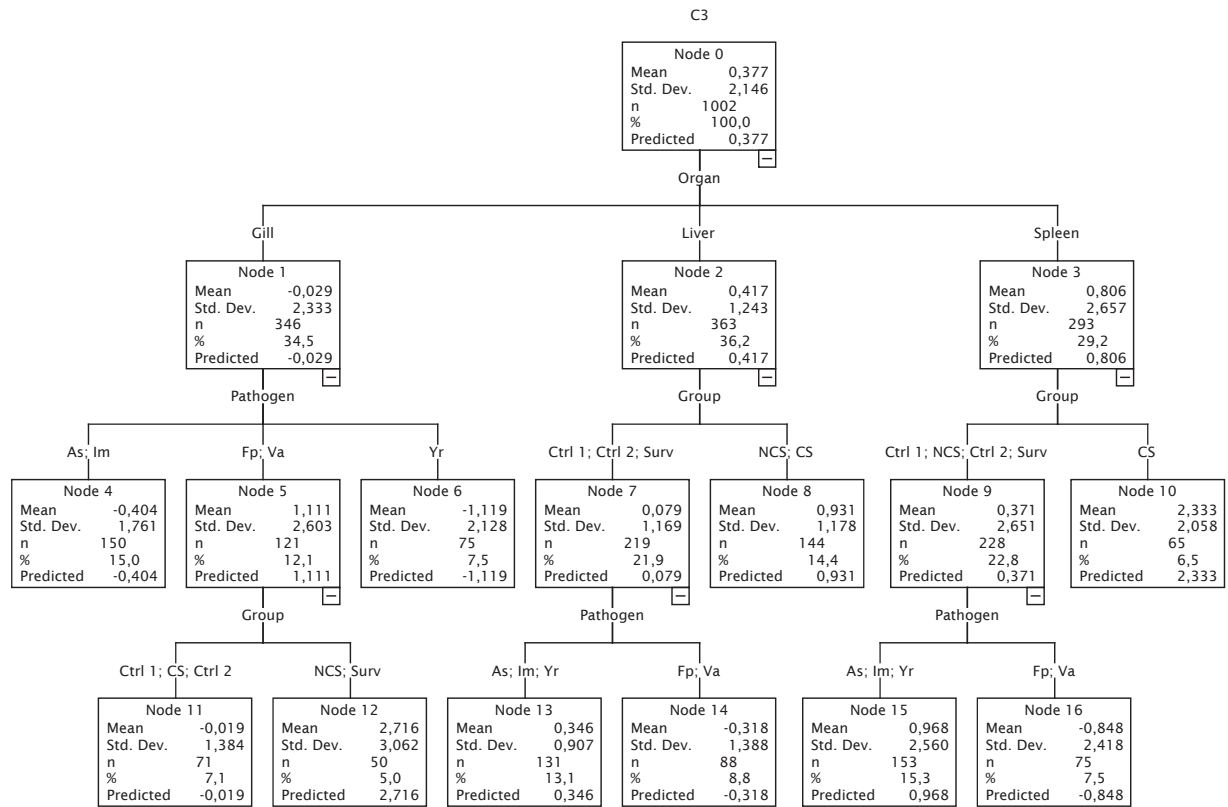

# Cathelicidin 1

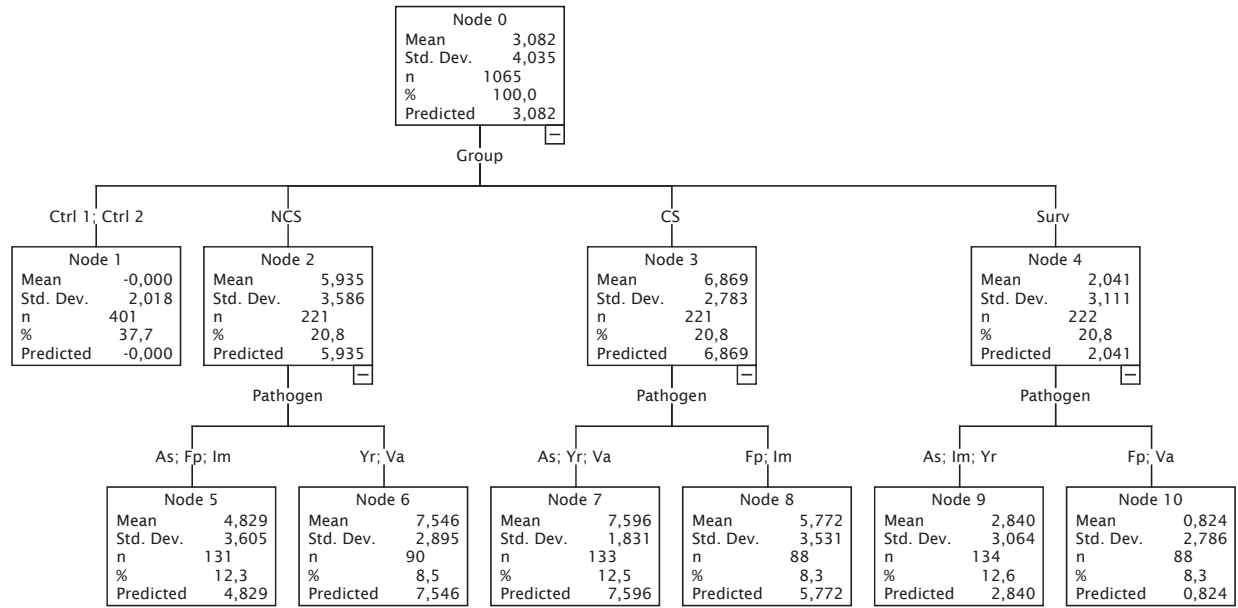

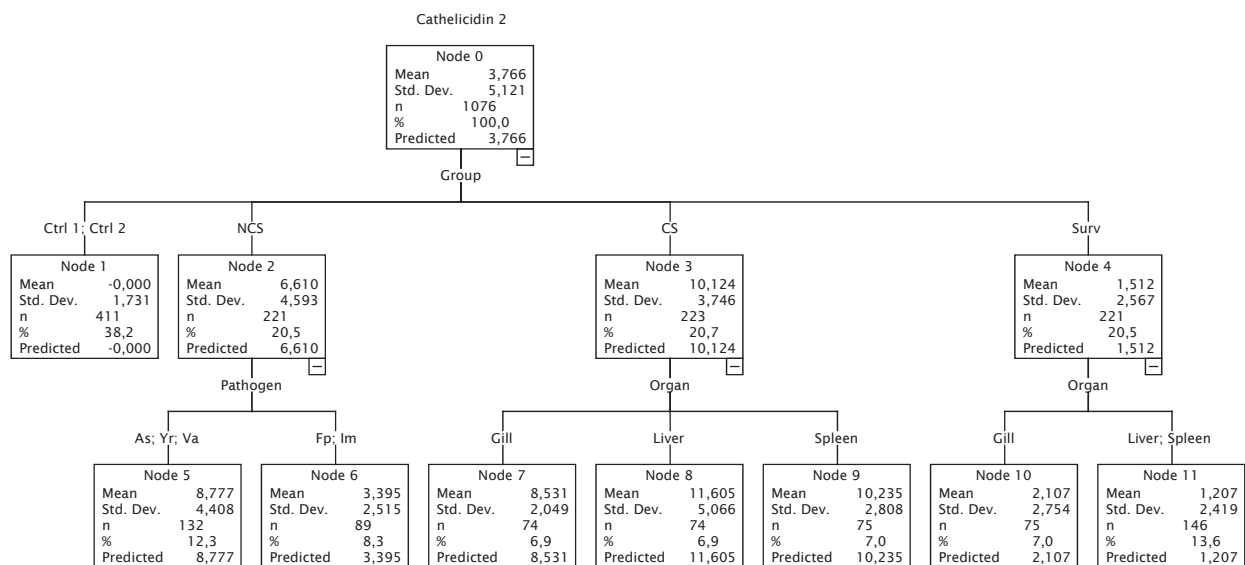

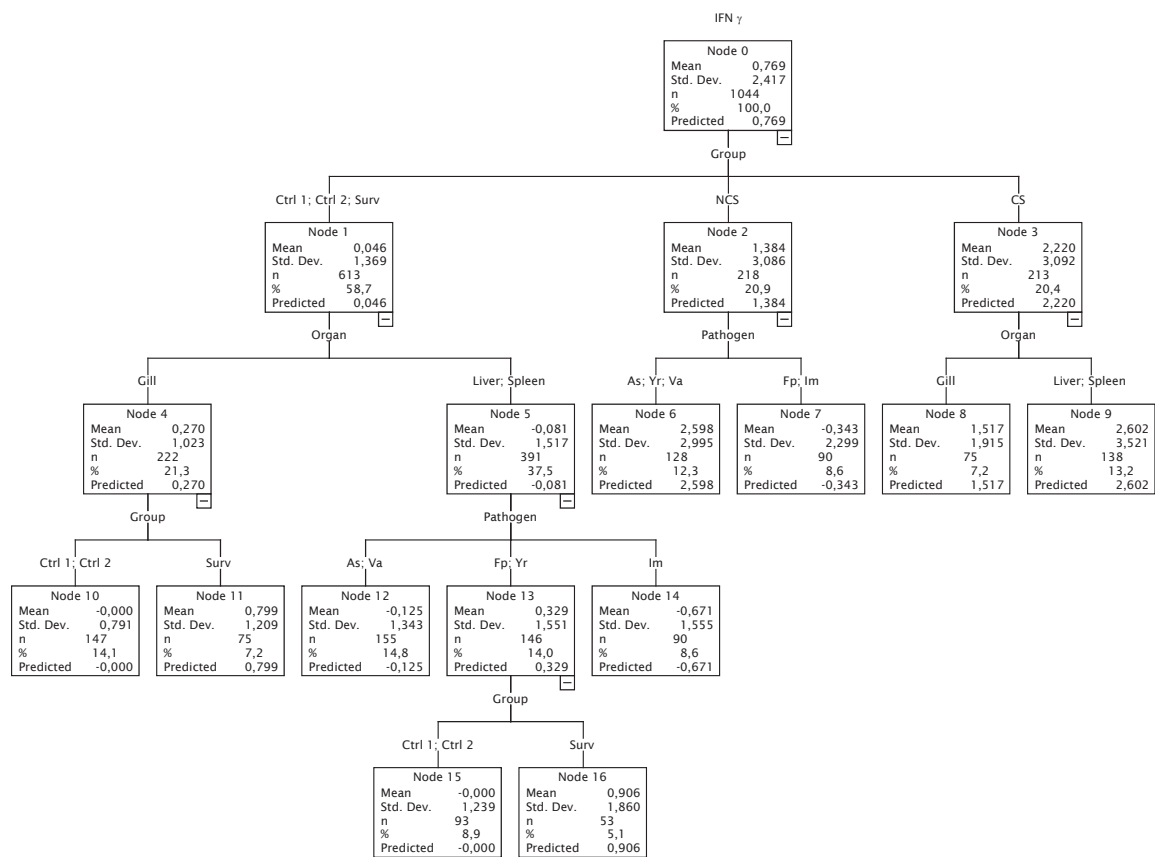

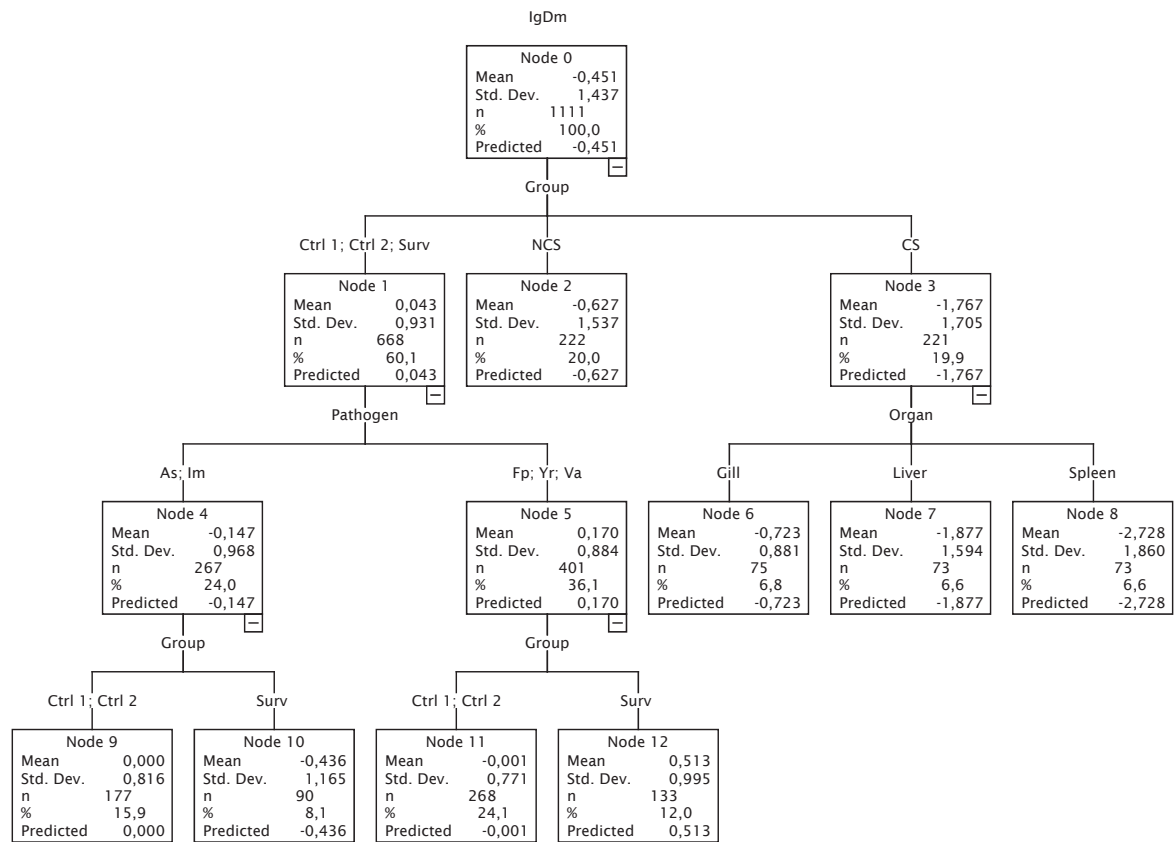

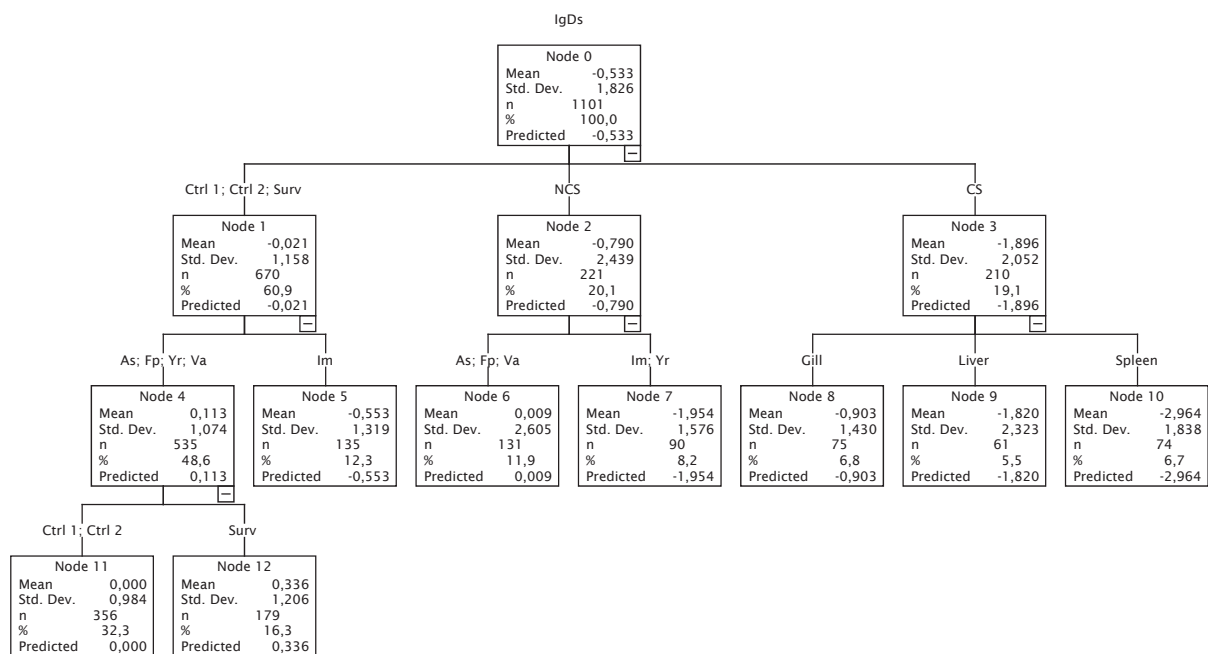

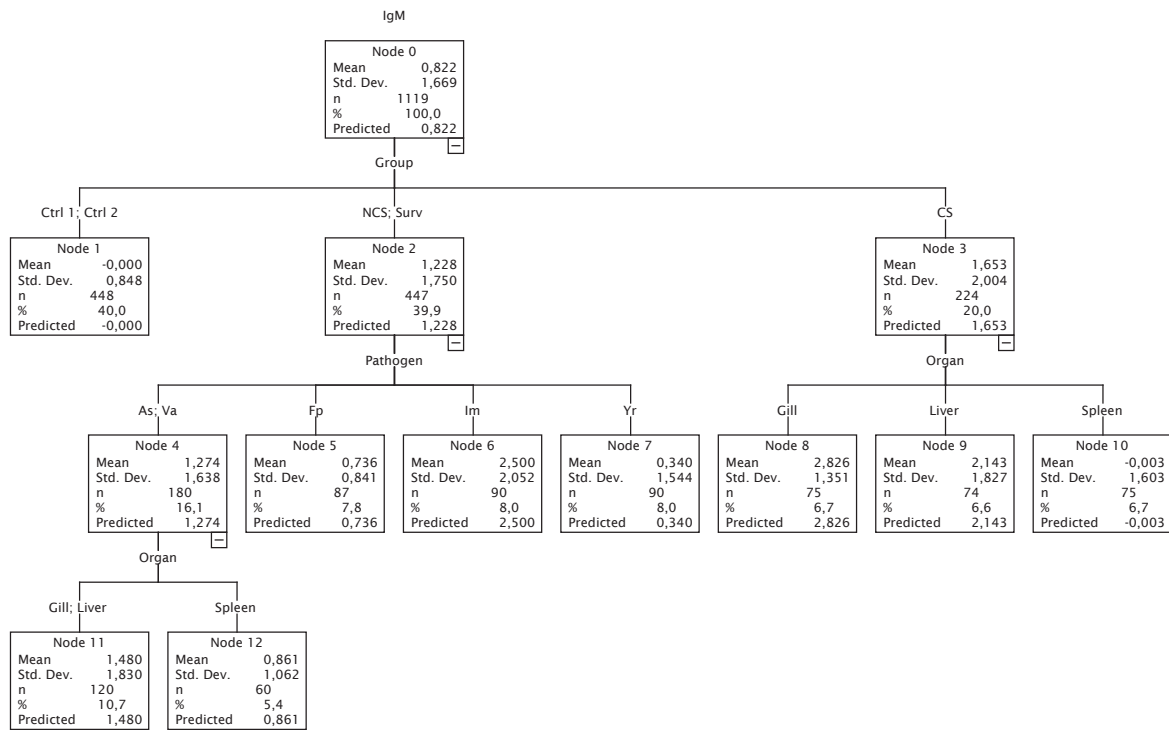

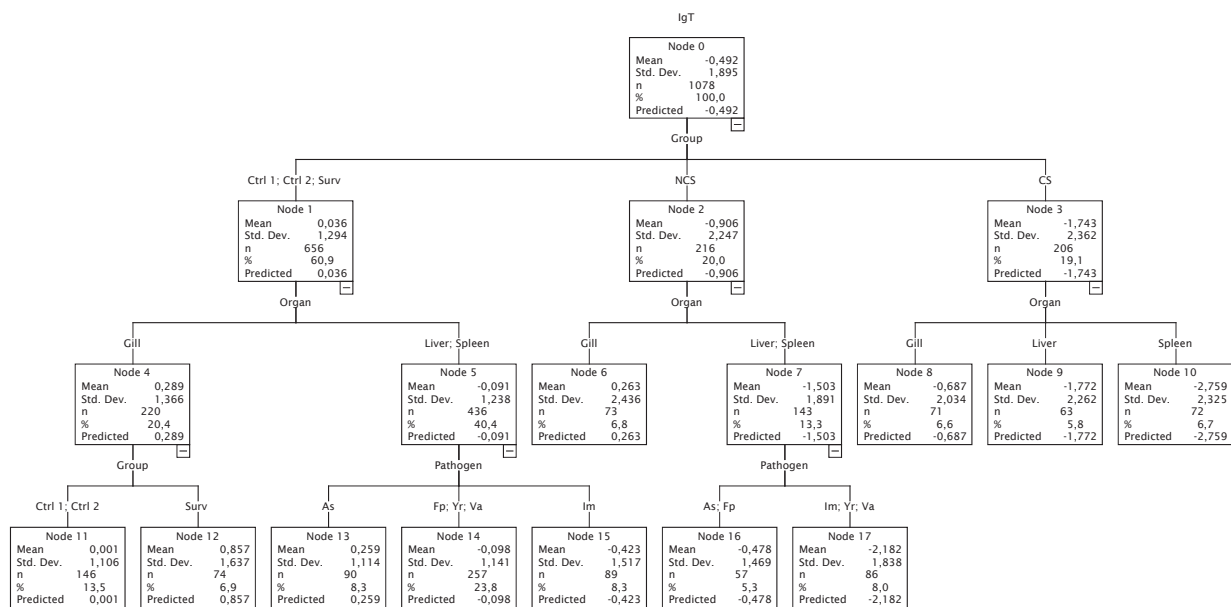

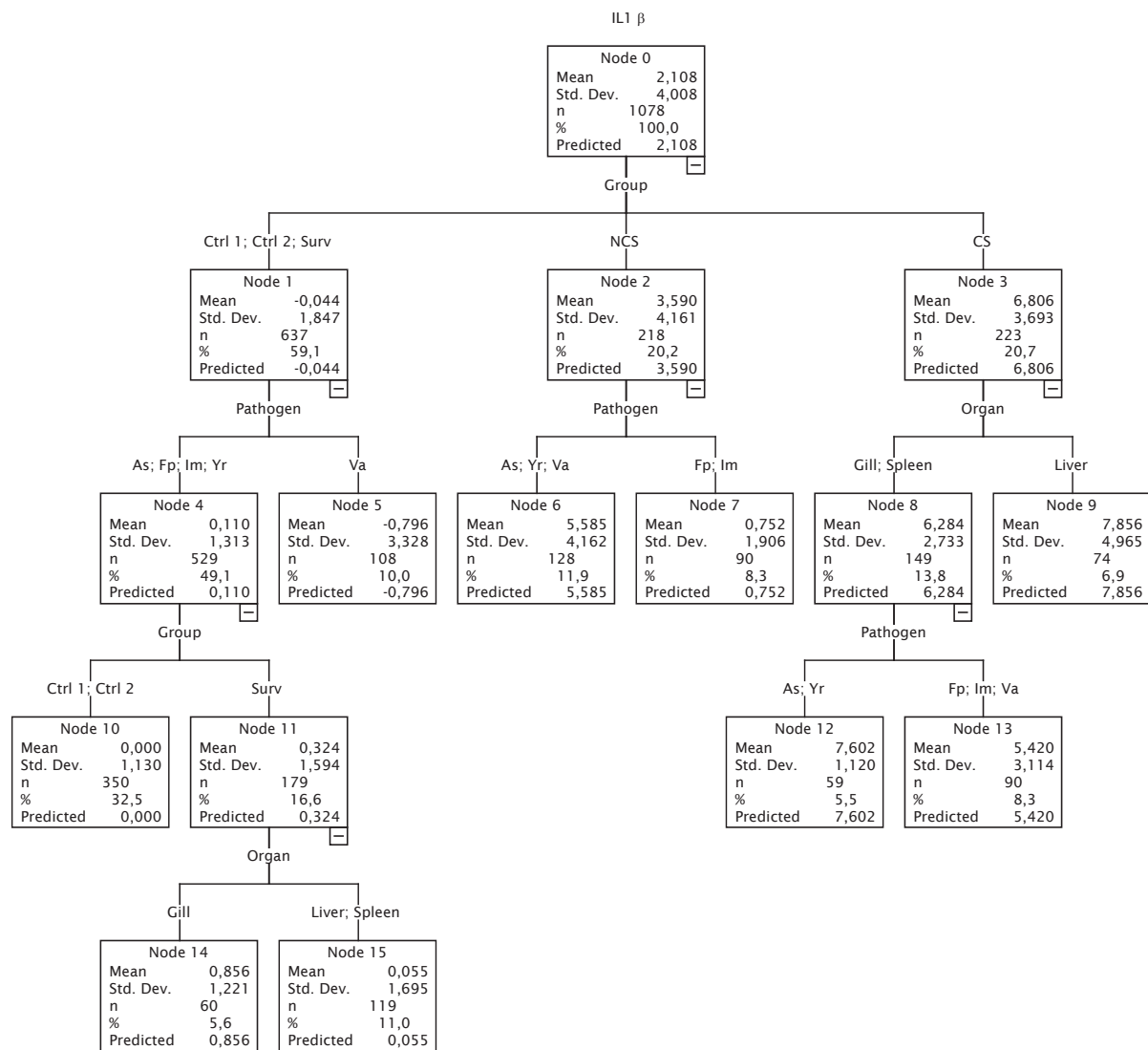

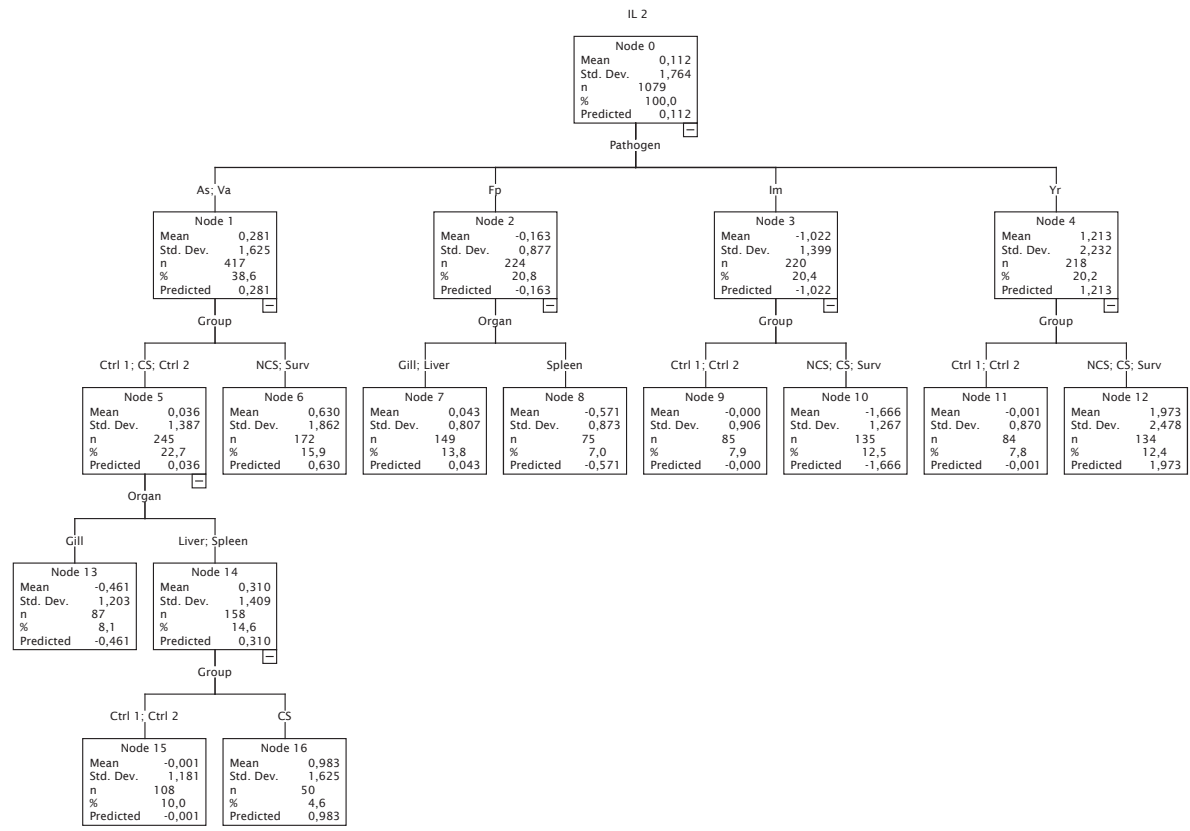

IL 4/13a

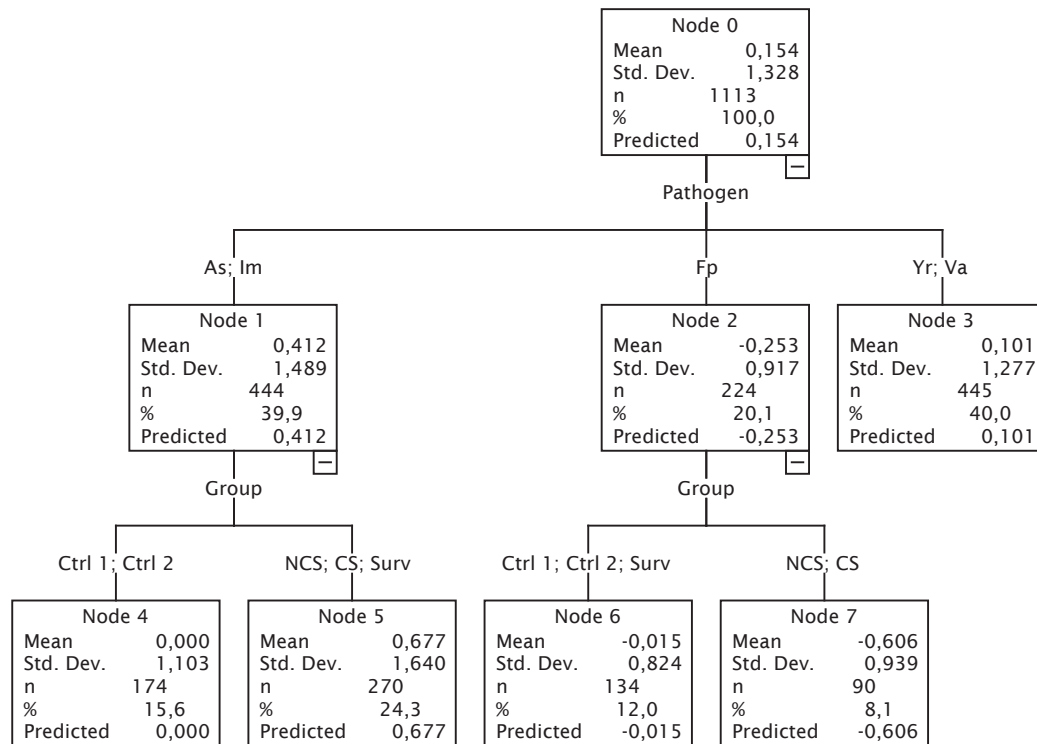

IL 6

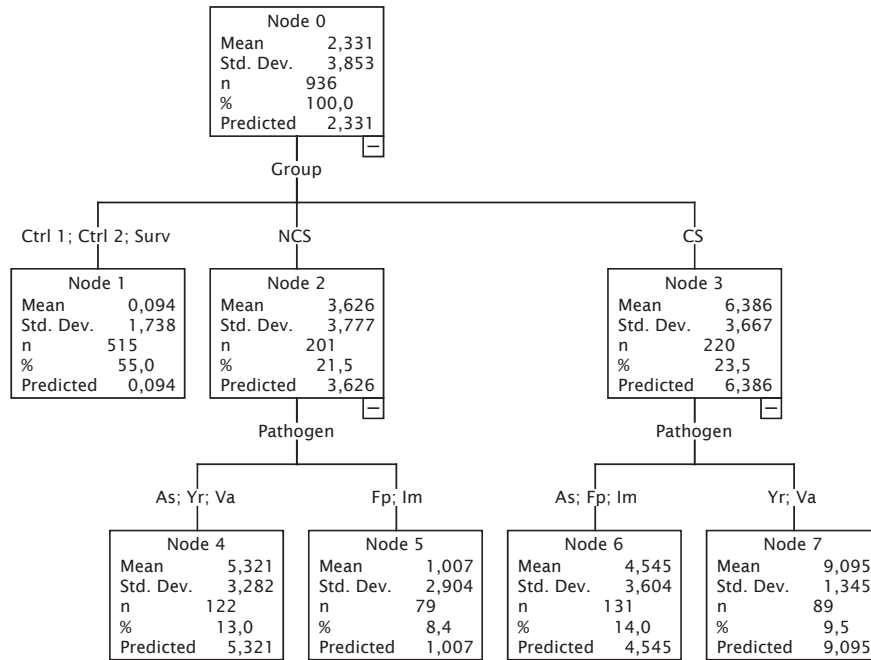

IL 8

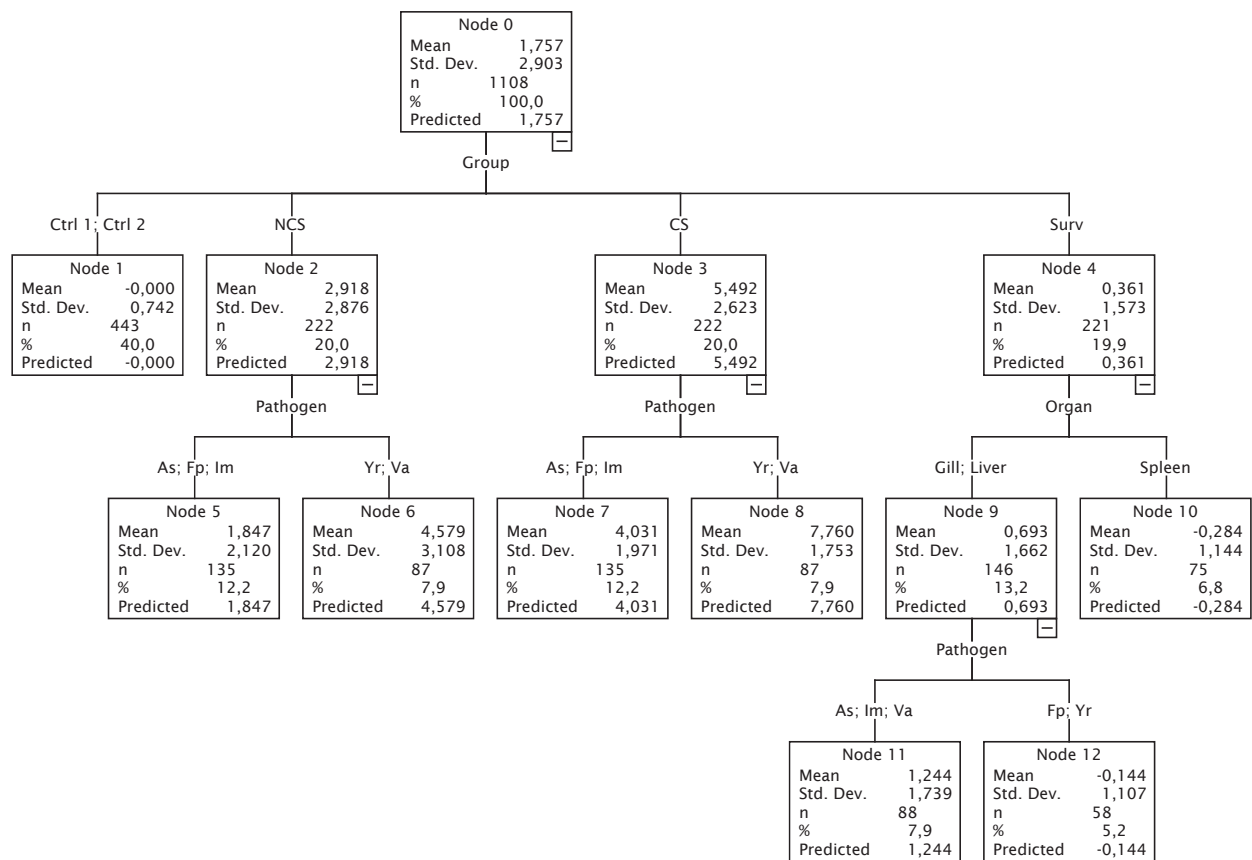

IL 10

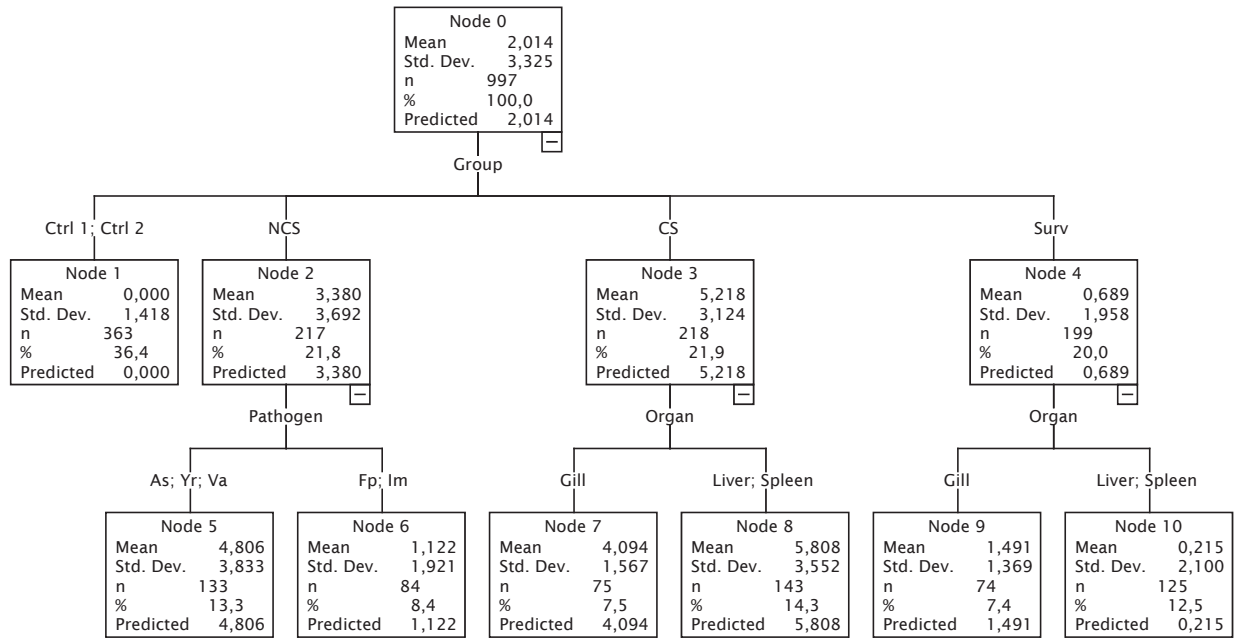

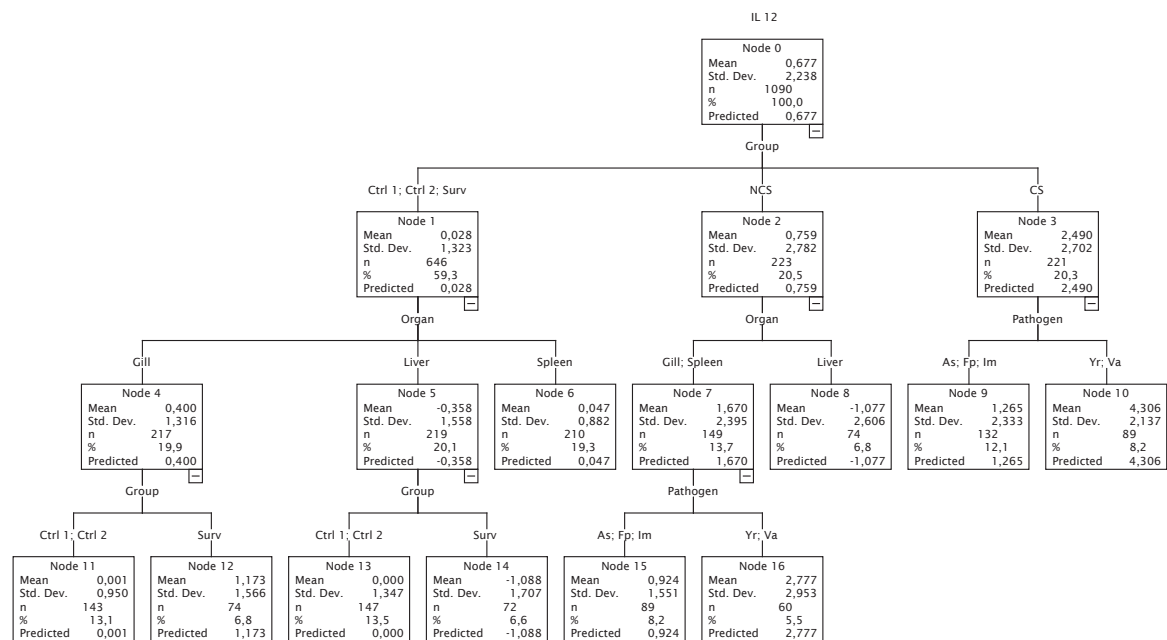

IL17 A/F2

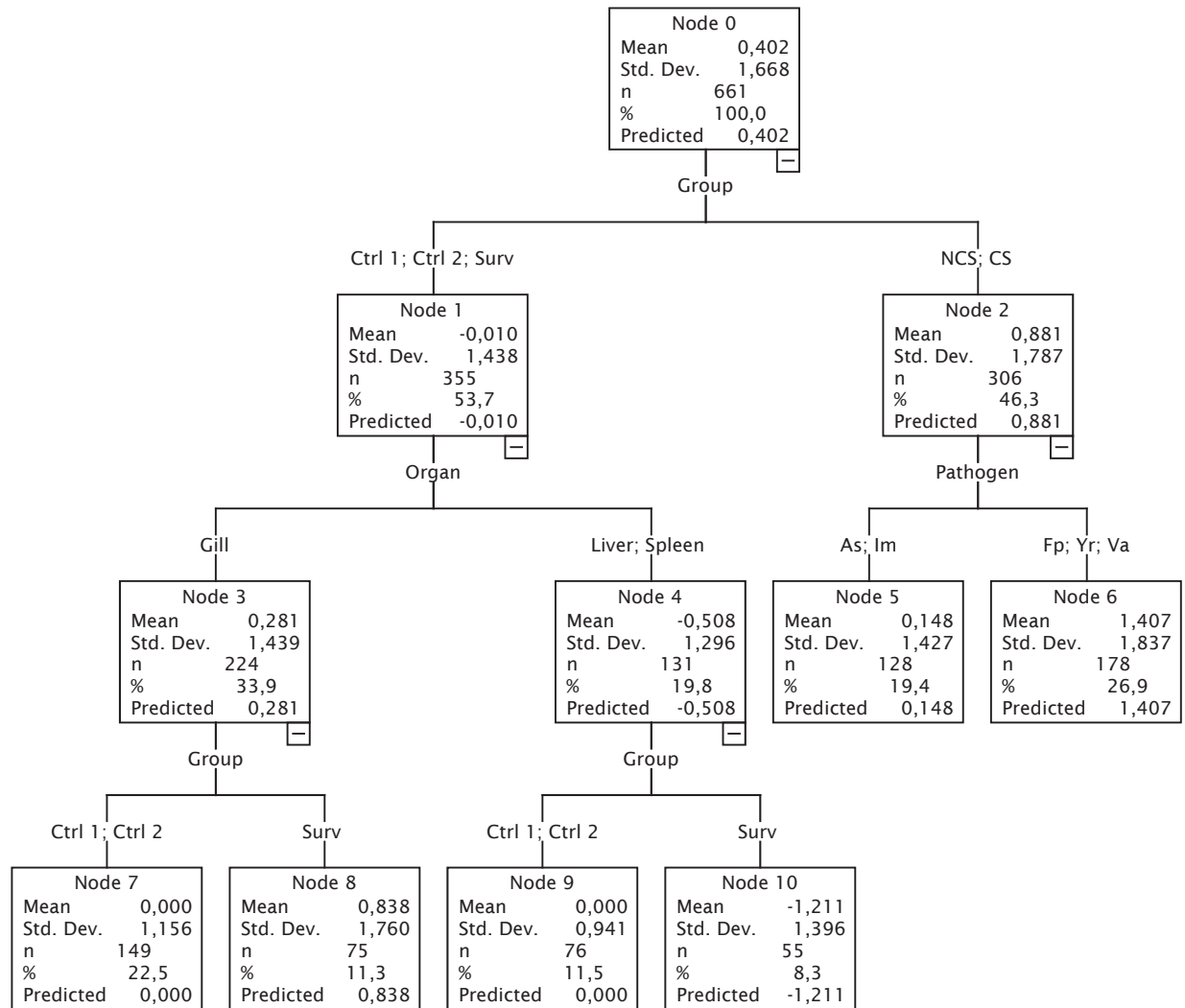

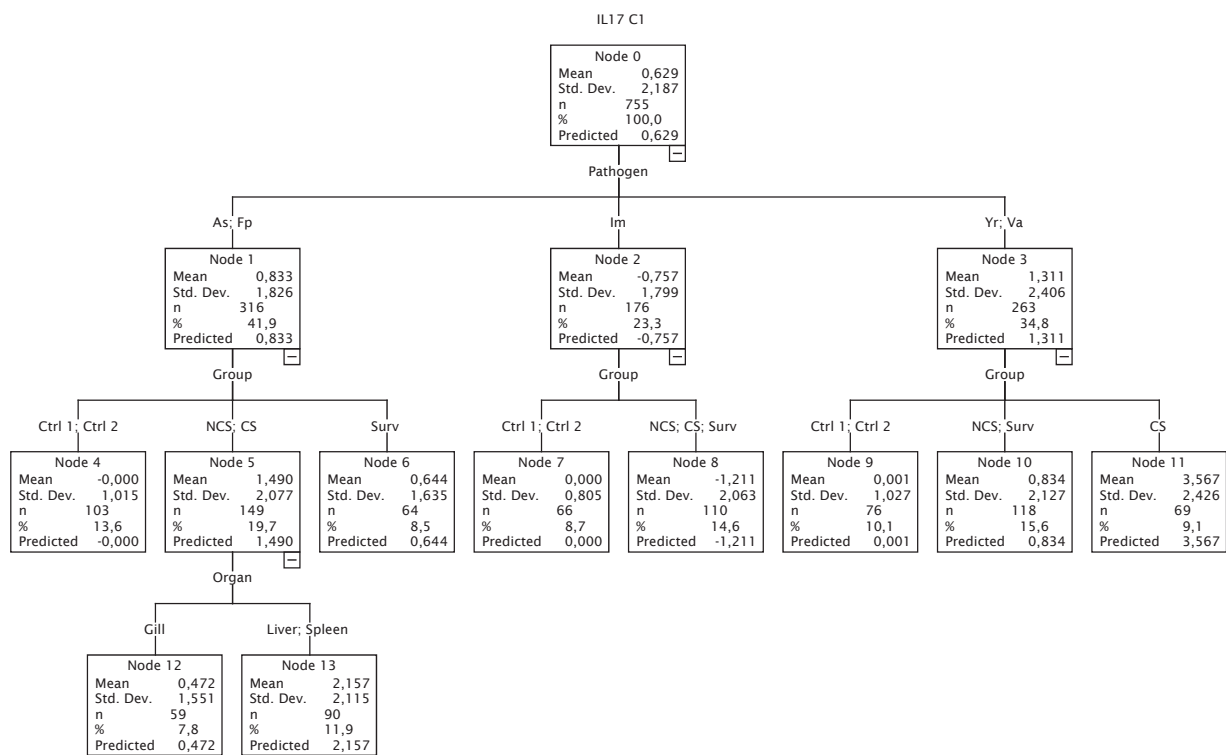

IL 17 C2

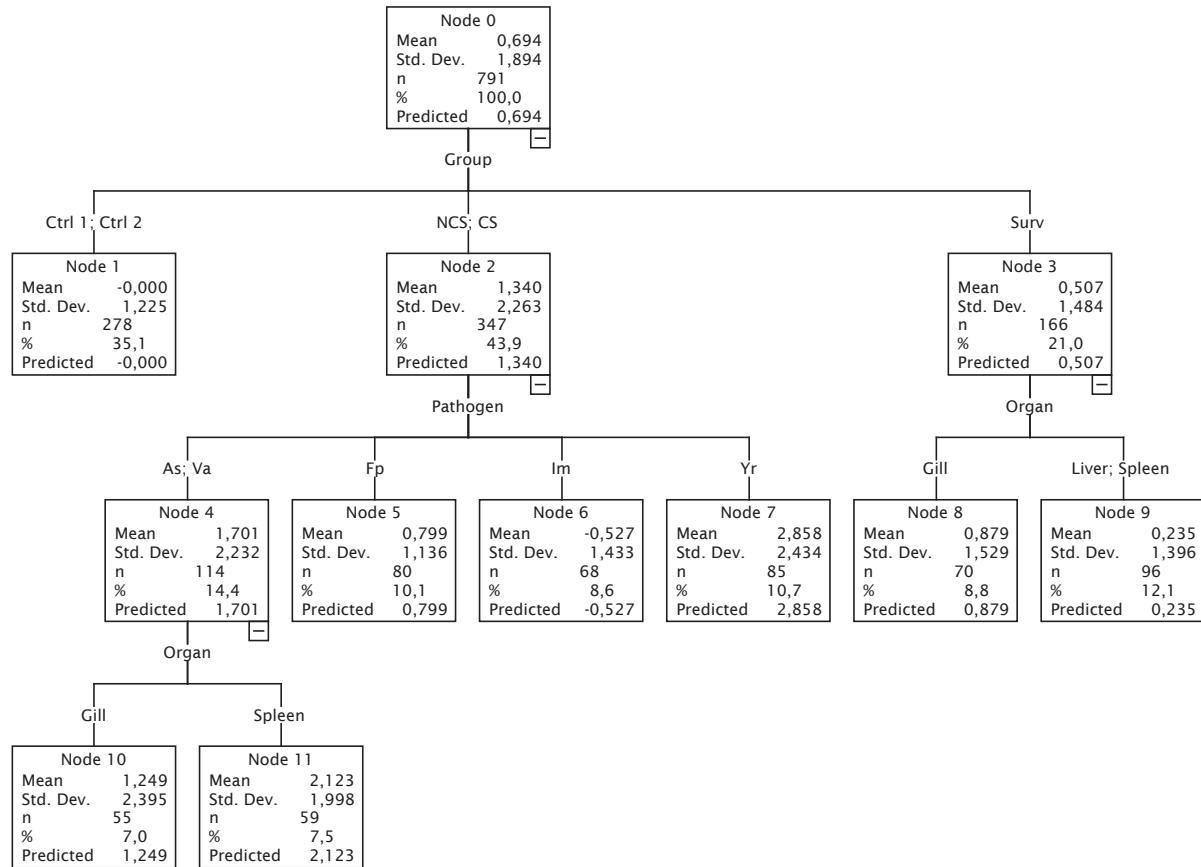

IL 22

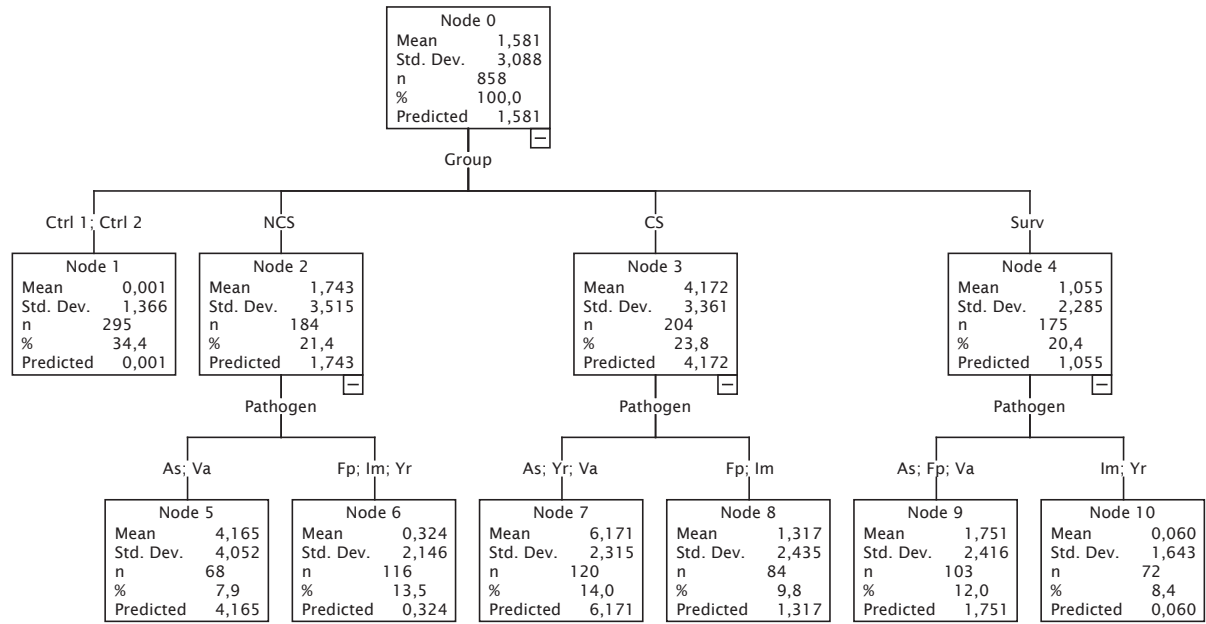

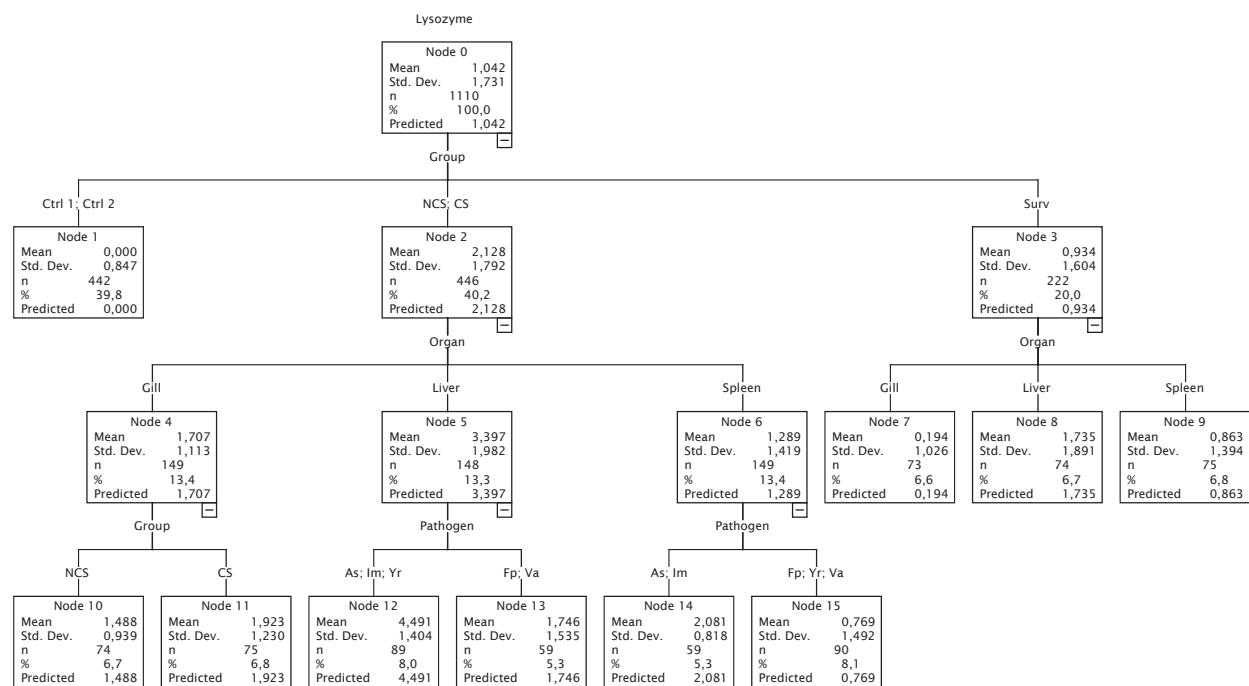

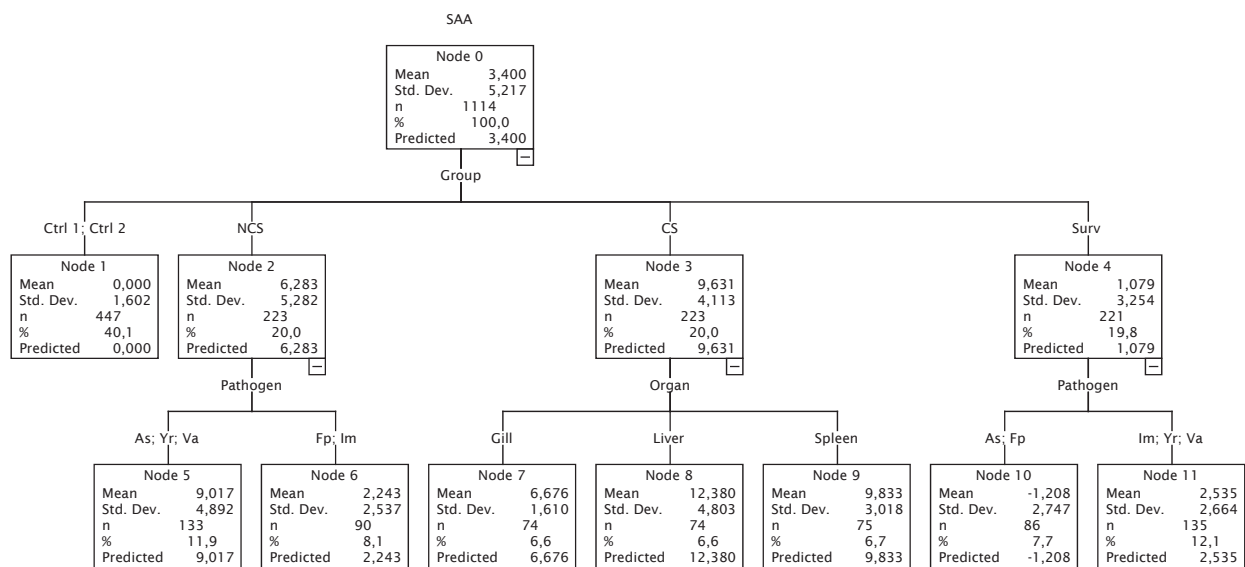

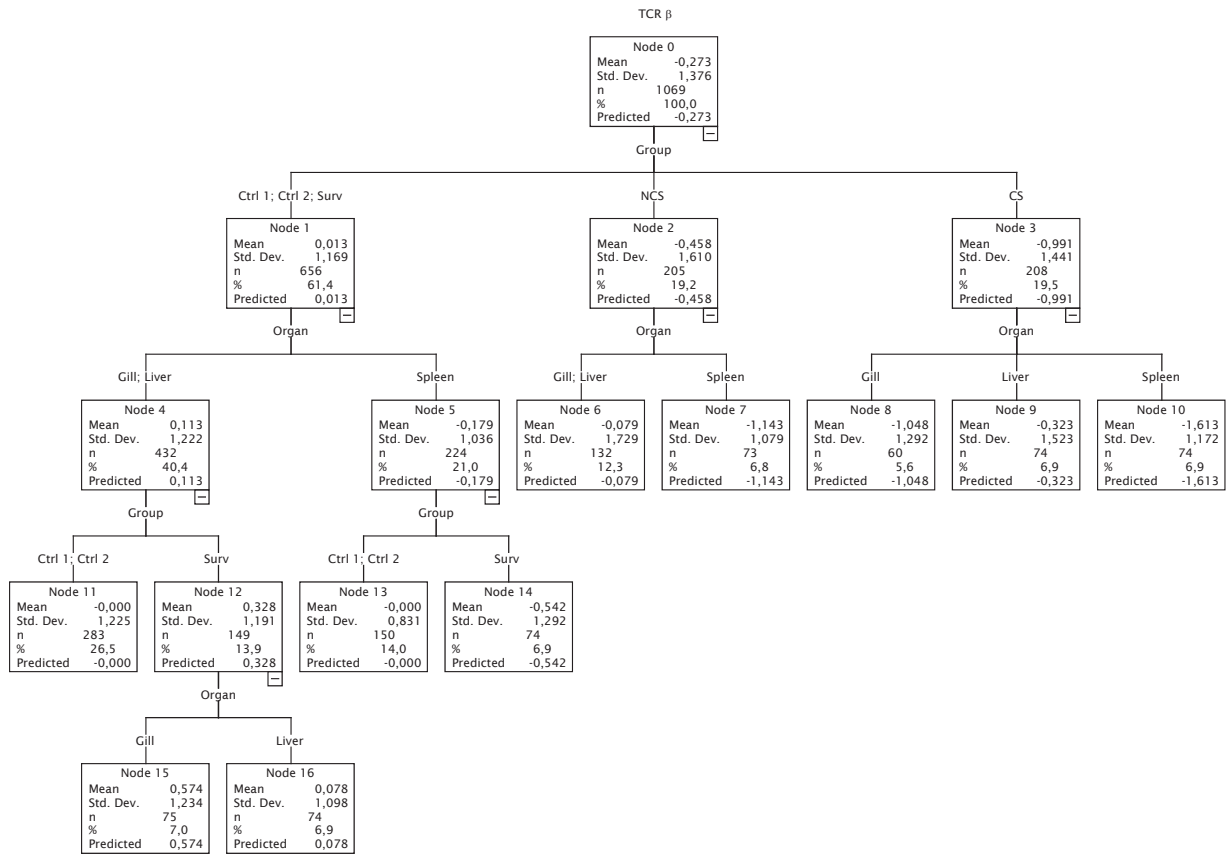

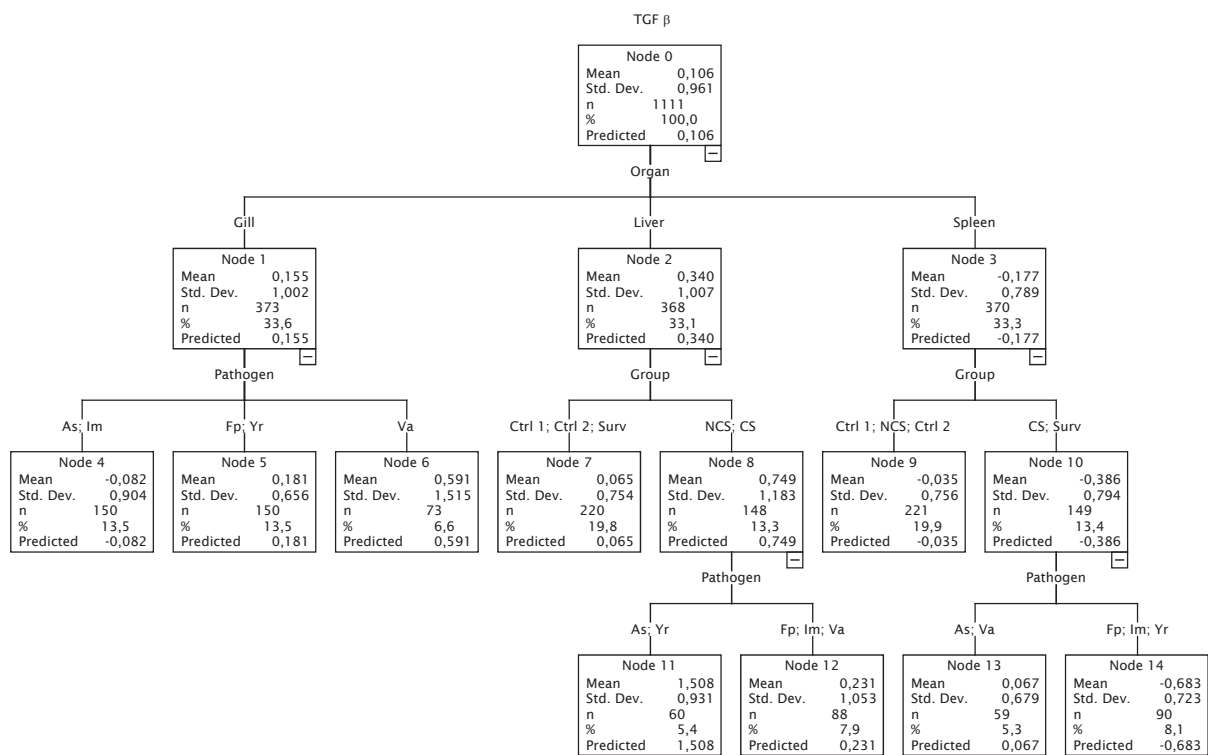

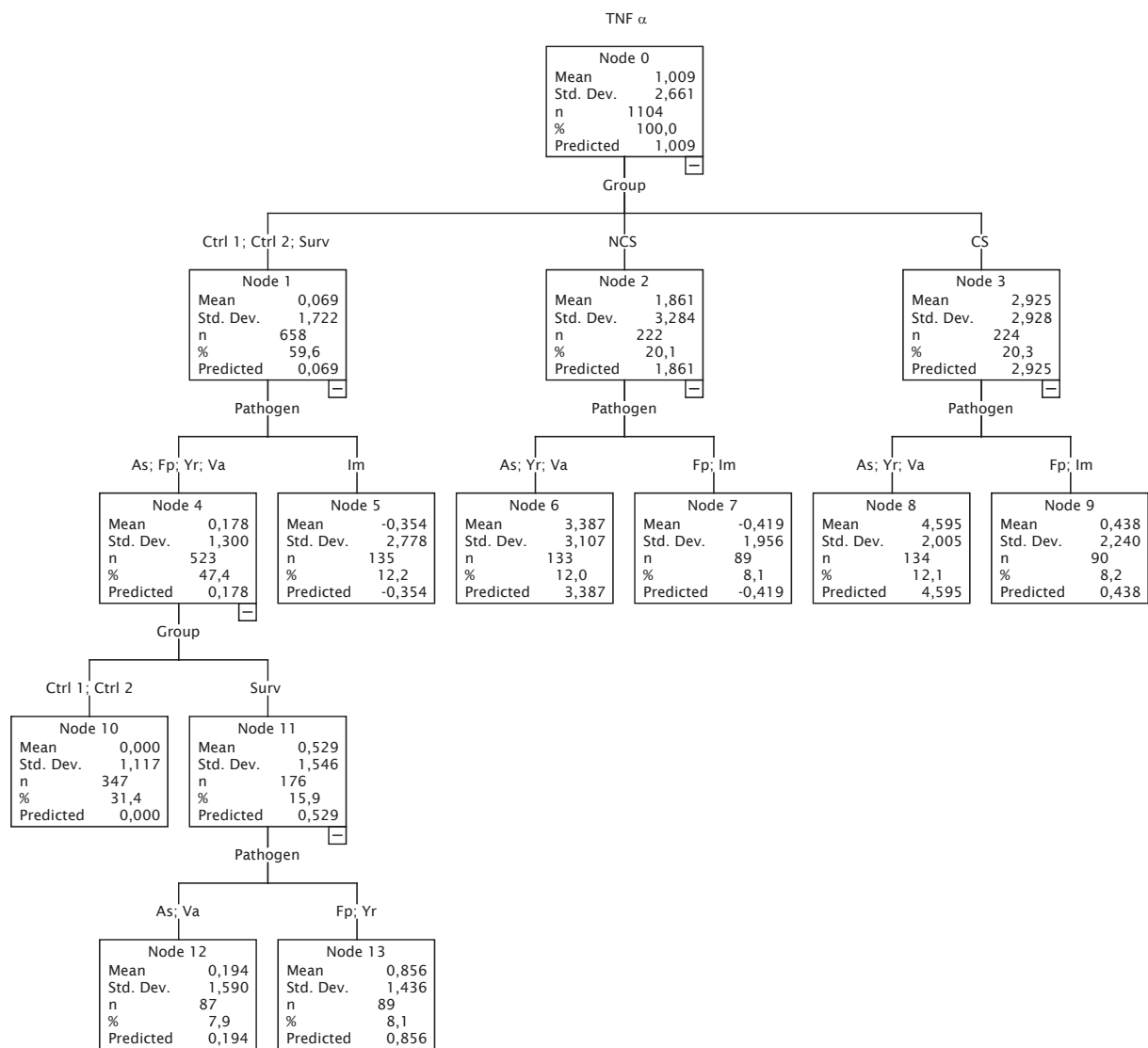

Supplement: Supplementary file 4 — Supplementary material file 4a. Classification trees. [file mmc4.pdf]
