# Supplementary material for: Differential immune gene expression in rainbow trout, Oncorhynchus mykiss (walbaum), exposed to five pathogens: Aeromonas salmonicida, Flavobacterium psychrophilum, Vibrio anguillarum, Yersinia ruckeri and Ichthyophthirius multifiliis
Source: Comp Immunol Rep. 2024 Sep 12;7:200166. doi: 10.1016/j.cirep.2024.200166 (PMC11437762; doi:10.1016/j.cirep.2024.200166)
Supplement: Supplementary file 5 — Supplementary material file 4b. Classification trees, description [file mmc5.docx]

*Classification trees*

The pathways involving various patterns of cytokine expression in the different host-pathogen systems are illustrated by the branching of the classification trees (Suppl. File S4 (*S4_Classification trees .pdf*). An overview is provided in Table 3

Primary separations of branches were all clean as they comprised of either Sampling groups (18 cases), Pathogens (3 cases), or Organs (3 cases). In case of the three genes IL-2, IL-4/13a, and IL-17C1 (page 10, 11 and 17, respectively), the pathogens showed to be the primary variable interacting with these genes. For expression of the IL-2 gene, AS grouped together with VA, FP, whereas YR, and ICH formed individual branches. Regarding IL-4/13a, FP formed an individual separation, AS and ICH constituted a second separation, and VA together with YR a third separation. In IL-17C1, ICH formed its own branch, another consisted of AS and FP together and a third YR and VA. The organs (gill, liver, and spleen) showed to be the primary variables when analyzing the genes encoding C3, IL-12 and TGF ß (Supplementary material XX page 1, 15 and 23, respectively). For the remaining 18 genes, the Sampling groups (NCS, CS, Surv, Controls) were the primary variables for interaction.

Secondary and tertiary separations of branches. Only in the case of TNF α (page 24), the pathogens solely constituted the secondary separation. For three of the genes (IgT, Lysozyme and TGF ß (page 8, 20 and 23, respectively), the pathogens were part of the tertiary or lower separation. In the case of TCR ß (page 22), there was no significant interaction of the pathogens. In the rest of 18 genes, the pathogens were part of the secondary separation; please note that for the genes mentioned, tertiary and below positions might also occur under treatment groups and organs not mentioned in the third column of table 3.

**Table 3. An** **overview of the independent variable Pathogen’s interaction in the classification trees.** 24 trees (supplementary material Xx), one for each gene, were constructed using the non-parametric growing method CHAID; branches were collapsed when p≥0.05. ^a^ indicates which branch the secondary separation occurred; for the indicated genes, and organs not indicated in the preceding column. ^b^ here genes for which no separation was evident above the tertiary separation is presented.

| Primary separation | Secondary separation: | under the primary branch ^a^ | Tertiary separation and below ^b^ |
| --- | --- | --- | --- |
| IL 2 | TNFα | NCS & CS & Surv & Ctrl | C3 |
| IL 4/13A | IL 17 C2 | NCS & CS & Surv | IgT |
| IL 17 C1 | IL 22 | NCS & CS & Surv | Lysozyme |
|  | IgDs | NCS & CS & Ctrl |  |
|  | Cath 2 | NCS & CS |  |
|  | IL 6 | NCS & CS |  |
|  | IL 10 | NCS & CS |  |
|  | IL 17A/F2 | NCS & CS |  |
|  | IgDs | NCS & Surv & Ctrl |  |
|  | IL 1ß | NCS & Surv & Ctrl |  |
|  | IL 8 | NCS & Surv & Ctrl |  |
|  | SAA | NCS & Surv & Ctrl |  |
|  | IgM | NCS & Surv |  |
|  | Cath 1 | NCS |  |
|  | INFg | NCS |  |
|  | IL12 | CS |  |
|  | IgDm | Surv & Ctrl |  |
|  | TGF ß | Gill |  |
