# Supplementary material for: Differential immune gene expression in rainbow trout, Oncorhynchus mykiss (walbaum), exposed to five pathogens: Aeromonas salmonicida, Flavobacterium psychrophilum, Vibrio anguillarum, Yersinia ruckeri and Ichthyophthirius multifiliis
Source: Comp Immunol Rep. 2024 Sep 12;7:200166. doi: 10.1016/j.cirep.2024.200166 (PMC11437762; doi:10.1016/j.cirep.2024.200166)
Supplement: Supplementary file 7 — Supplementary material file 6. Pc score plots showing the contribution of the sampling groups to the Component plot Fig. 2c. [file mmc7.pdf]

Supplemental File S6. PC score plots of sampling groups.

Illustrations of the contributions of the sampling groups (Clinical Sign (CS), No Clinical Signs (NCS), Survivor (Surv), and uninfected control groups) to the distribution of genes seen in component plots in Fig. 2. PC indicates principal component.

On the next four pages, 4 panels:

1. **Suppl. File S6a.** PC score plot of PC1 vs PC2.
2. **Suppl. File S6b.** PC score plot of PC1 vs PC3.
3. **Suppl. File S6c.** PC score plot of PC2 vs PC3.
4. **Suppl. File S6d.** Bar plots of the contributions of the sampling groups to the three first principal components.

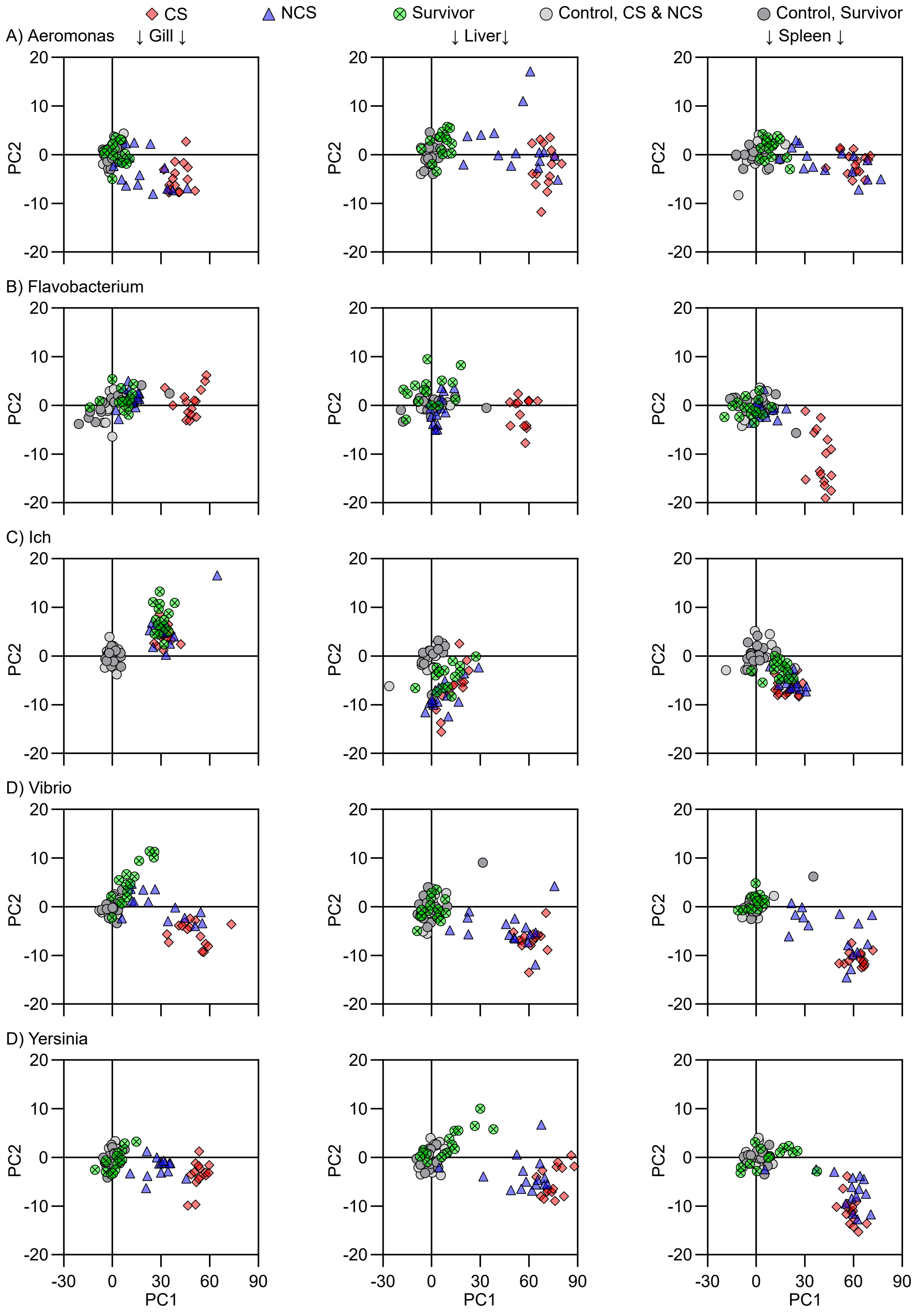

Supplemental File S6a. PC score plots of sampling groups in the sampling groups. PC1 vs PC2. Illustration of the contribution of the sampling groups (Clinical Sign (CS), No Clinical Signs (NCS), Survivor (Surv), and uninfected control groups) to the distribution of genes seen in component plots in Fig. 2. PC indicates principal component.

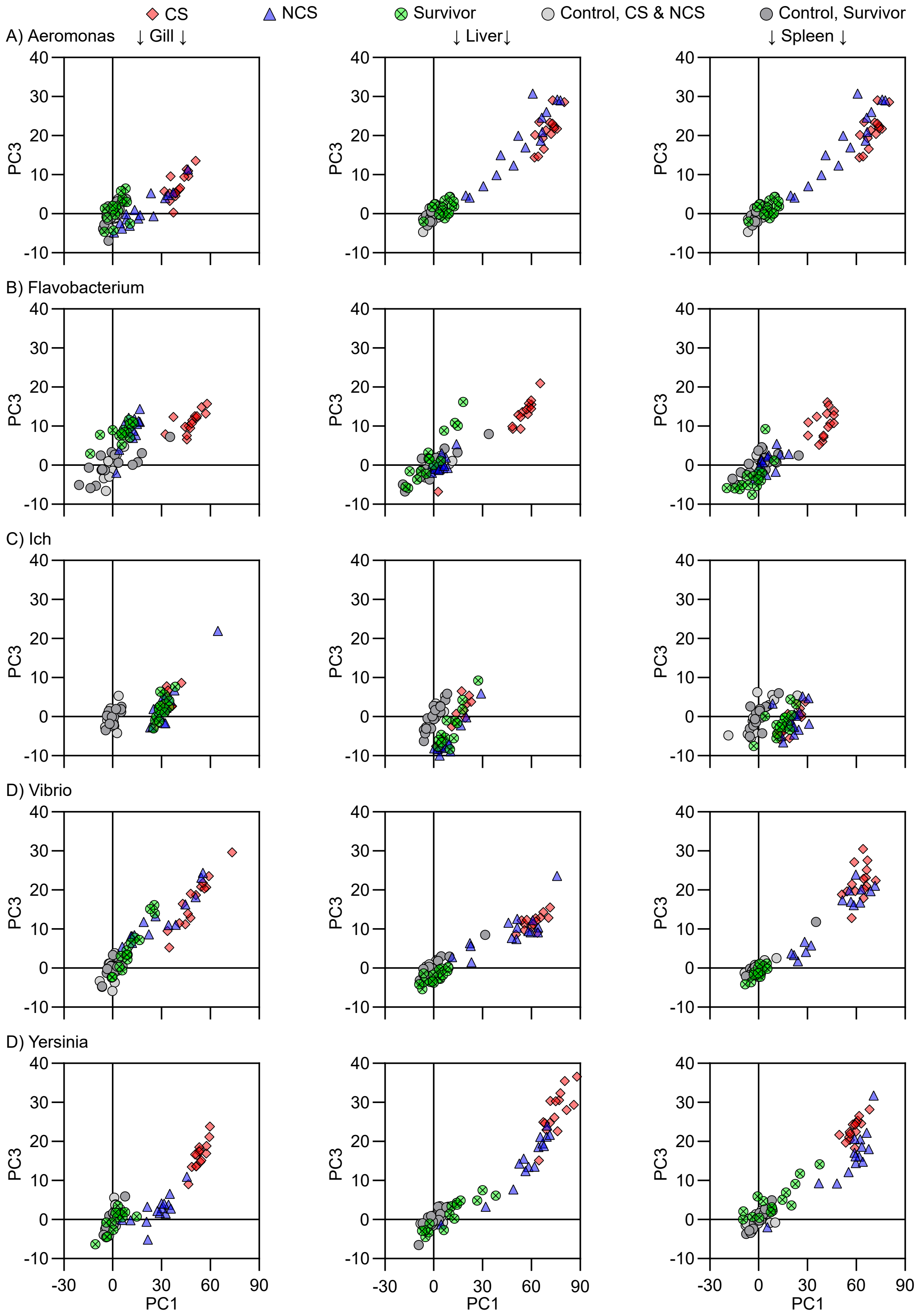

Supplemental File S6b. PC score plots of sampling groups in the sampling groups. PC1 vs PC3. Illustration of the contribution of the sampling groups (Clinical Sign (CS), No Clinical Signs (NCS), Survivor (Surv), and uninfected control groups) to the distribution of genes seen in component plots in Fig. 2. PC indicates principal component.

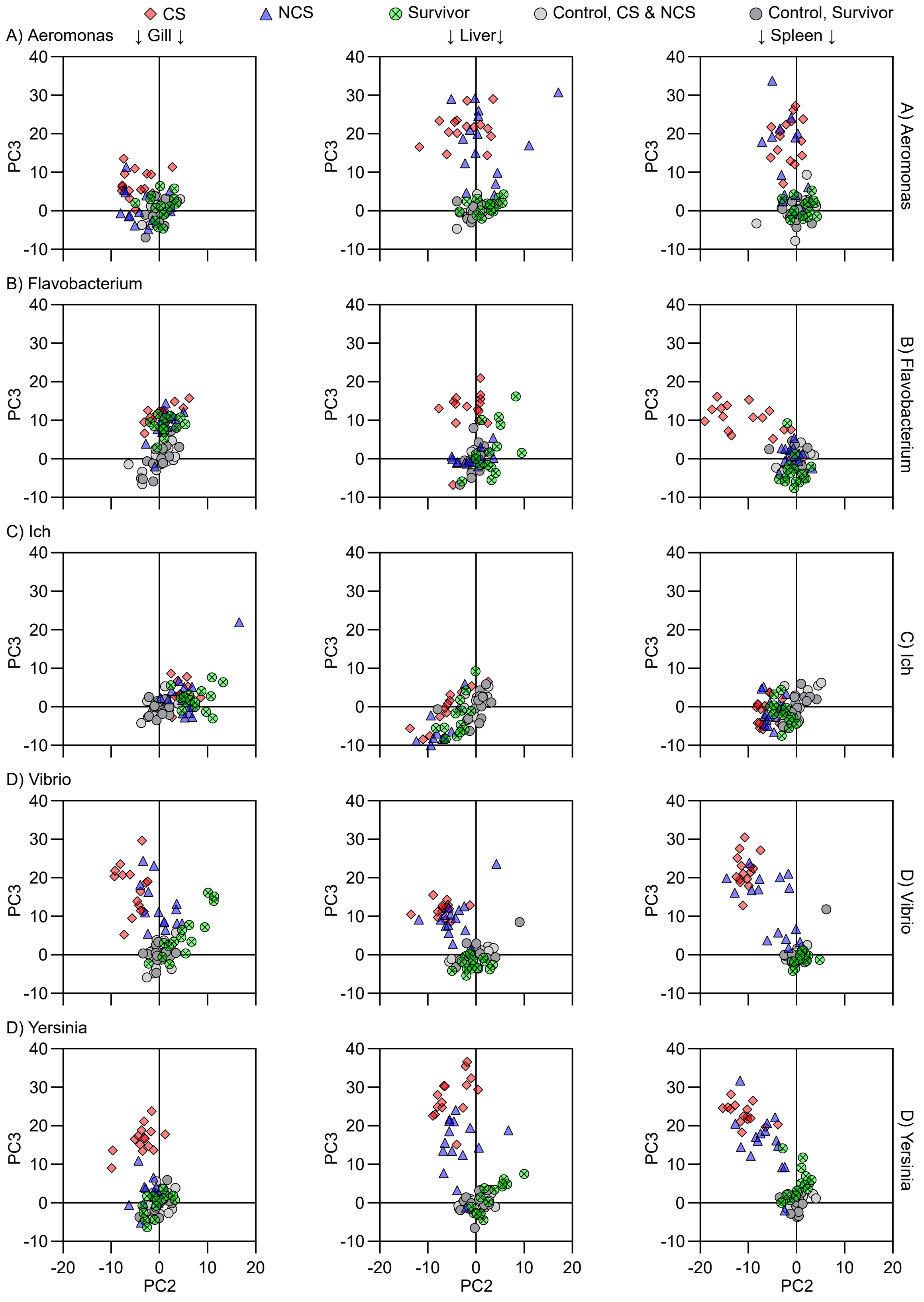

Supplemental File S6c. PC score plots of sampling groups in the sampling groups. PC2 vs PC3. Illustration of the contribution of the sampling groups (Clinical Sign (CS), No Clinical Signs (NCS), Survivor (Surv), and uninfected control groups) to the distribution of genes seen in component plots in Fig. 2. PC indicates principal component.

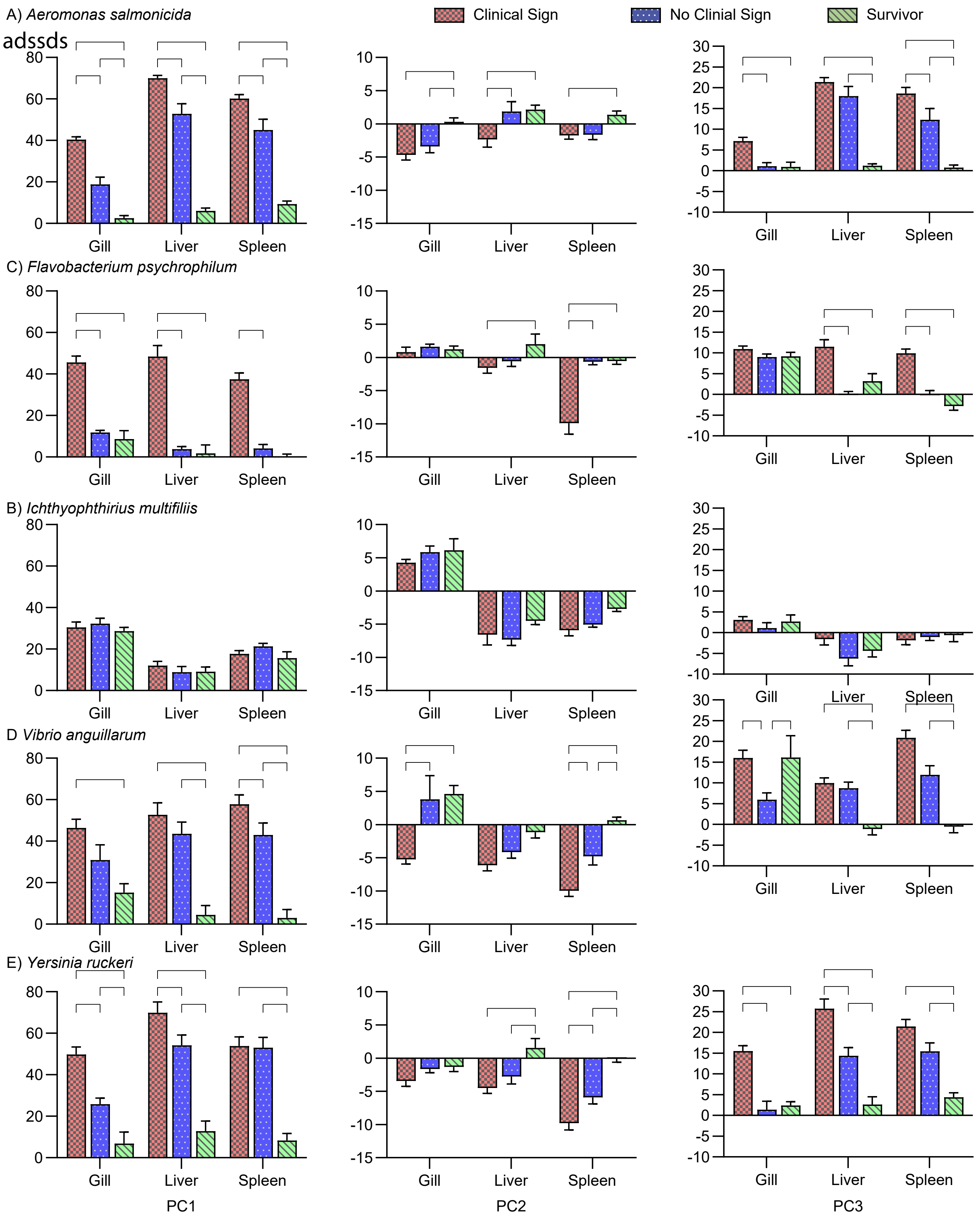

S6d. Bar plots showing the contribution to the component plot (Fig. 2) of the infected sampling groups. Brackets indicate significant differences between groups when tested using one-way ANOVA with Tukey's multiple comparisons test and p<0.05. Se Suppl. File S7d for significant differences betweeen organs. The primary drivers of PC1, PC1, and PC3 were Innate, Th2, and Th17 groups of genes, respectively; the Th2 group was the secondary driver of all three PC's.
