# Supplementary material for: Differential immune gene expression in rainbow trout, Oncorhynchus mykiss (walbaum), exposed to five pathogens: Aeromonas salmonicida, Flavobacterium psychrophilum, Vibrio anguillarum, Yersinia ruckeri and Ichthyophthirius multifiliis
Source: Comp Immunol Rep. 2024 Sep 12;7:200166. doi: 10.1016/j.cirep.2024.200166 (PMC11437762; doi:10.1016/j.cirep.2024.200166)
Supplement: Supplementary file 9 [file mmc9.pdf]

Supplementary file 8. An overview of relative gene expression between infection groups (CS, NCS, and surv indicate clinical sign, no clinical sign, and survivors, respectively). significant ($p < 0.05$ and fold at least 2) Quantitative up and downregulations relative to uninfected controls groups are indicated by ↑ and ↓, respectively; # and ¤ indicate significant up- and downregulations of CS relative to NCS. In case of less than 3 samples in at least one of the groups compared, qualitative assessment based on presence/absence of Cq values was performed; here ▲ and ▼ indicate significant ($p < 0.05$) upregulation, downregulations relative to uninfected controls groups and NS indicate no significant qualitative regulations w. Pleiotropic genes, marked with an asterisk, have been reallocated according to results of Fig. 2b.

Grouping of genes→			Innate								Th1-like				Th2-like						Th17-like					
←Pathogen	←Group	Gene → Organ ↓	IL1ß	IL-8	IL-6*	IL-10*	Cathelicidin 1	Cathelicidin 2	Lysozyme	SAA	IFNγ	IL-12	TGFß*	TNFα	Il-4/13a	IgDm	IgDs	IgM	IgT	TCRß	C3	IL-2	IL-17A/F2	IL-17C1	IL-17C2	IL-22
Areomonas	CS	Gill	↑#	↑#	↑#	#	↑#	↑#	↑	↑#		#		#			↓					↓				↑#
		Liver	↑#	↑#	↑#	↑	↑#	↑#	↑	↑#	↑		↑	↑	↑		¤	↑				↑		▲	▼	↑
		Spleen	↑#	↑#	↑	↑#	↑	↑#	↑	↑#	↑	#		↑#				↑							↑	
	NCS	Gill	↑	↑	↑		↑	↑	↑	↑							↓									↑
		Liver	↑	↑	↑	↑	↑	↑	↑	↑	↑		↑	↑	↑			↑				↑		▲	▲	↑
		Spleen	↑	↑	↑	↑	↑	↑	↑	↑	↑	↑		↑				↑							↑	↑
	Surv	Gill			↓	↑		↑													↓		↑			↑
		Liver	↑	↑	NS				↑						↑			↑						▲	NS	
		Spleen					↑	↑	↑	↑								↑								
Flavobacterium	CS	Gill	↑#	↑#	↑#	#	↑#	↑#	↑	↑#		#						#								↑
		Liver	↑#	↑#	↑#	↑	↑#	↑#	↑	↑#	↑			↑				↑			↑			↑	↑	↑
		Spleen	↑#	↑#	↑	↑#	↑	↑#	↑	↑#										↓				▲		
	NCS	Gill	↑		↑	↑	↑	↑	↑					↑												↑
		Liver					↑	↑							↓											↑
		Spleen						↑																▲		
	Surv	Gill				↑						↑						↑			↑		↑	↑	↑	↑
		Liver		↓						↓	↑						↑									↑
		Spleen				↓				↓											↓		↓			
Ich	CS	Gill	↑	↑	↑		↑	↑		↑						↓				▼						↑
		Liver		↑	↓		↑	↑	↑	↑	↓			↓						↓		↓		↓		↓
		Spleen			↑	↑	↑	↑	↑	↑	↓			↓						↓			▲			
	NCS	Gill	↑	↑	↑	↑	↑	↑		↑		↑			↑			↑		▼						↑
		Liver	↓	↑	↓	↓	↑	↑	↑	↑	↓			↓					↓			↓		↓		↓
		Spleen	↑		↑	↑	↑	↑	↑	↑	↓												▲			
	Surv	Gill	↑	↑	↑	↑	↑	↑		↑	↑	↑			↑			↑	↑			↓				
		Liver	↓	↑	↓	↓	↑	↑	↑	↑	↓	↓		↓	↓	↓	↓	↑			↑	↓	NS	↓		↓
		Spleen				↑	↑	↑	↑	↑	↓		↓	↓	↑		↓	↑	↓	↓	↑	↓	▲	NS		
Vibrio	CS	Gill	↑#	↑#	↑#		↑#	↑#	↑	↑#				#		↓¤	↓					¤				↑#
		Liver	↑¤	↑#	▲	▲	↑	↑#	↑	↑#				↑			¤	↑					NS	▲	▲	▲
		Spleen	↑	↑#	↑	↑#	↑	↑#		↑#	↑	#		↑						↓			NS	▲	↑	
	NCS	Gill	↑	↑	↑	↑	↑	↑	↑	↑		↑		↑			↑	↑				↑				↑
		Liver	↑	↑	▲	▲	↑	↑	↑	↑	↑			↑					↓				NS	▲	▲	▲
		Spleen	↑	↑	↑	↑	↑	↑		↑	↑	↑		↑									NS	▲	↑	↑
	Surv	Gill		↑	↑	↑	↑	↑			↑	↑		↑	↑	↑	↑	↑		↑		↑			↑	↑
		Liver			NS	NS						↓									↓		NS	NS	NS	NS
		Spleen	↓																				NS	NS		
Yersinia	CS	Gill	↑#	↑#	↑#	#	↑#	↑#	↑	↑#		#		#			↓					↑#				↑#
		Liver	↑#	↑#	↑#	↑	↑	↑#	↑	↑	↑		↑	↑	↑			↑				↑	▲	↑	↑	↑
		Spleen	↑	↑#	↑	↑	↑	↑		↑	▲	#		↑¤				↓					▲	▲	↑	▲
	NCS	Gill	↑	↑	↑	↑	↑	↑	↑	↑		↑						↑				↑				
		Liver	↑	↑	↑	↑	↑	↑	↑	↑	↑		↑	↑				↑				↑	▲	↑	↑	
		Spleen	↑	↑	↑	↑	↑	↑	↑	↑	▲	↑		↑									▲	▲	↑	▲
	Surv	Gill																			↓					
		Liver					↑		↑					↑		↑							NS			
		Spleen					↑	↑		↑				↑							↑		▲	▲		
