# Supplementary material for: Differential immune gene expression in rainbow trout, Oncorhynchus mykiss (walbaum), exposed to five pathogens: Aeromonas salmonicida, Flavobacterium psychrophilum, Vibrio anguillarum, Yersinia ruckeri and Ichthyophthirius multifiliis
Source: Comp Immunol Rep. 2024 Sep 12;7:200166. doi: 10.1016/j.cirep.2024.200166 (PMC11437762; doi:10.1016/j.cirep.2024.200166)
Supplement: Supplementary file 10 [file mmc10.pdf]

S9. Clinical signs. gene expression analysis comparing the pathogens to each other's. Significant quantitative up- and downregulations (fold at least 2 and $p < 0.5$ of one-way ANOVA) relative to reference pathogens are indicated by ↑ and ↓, respectively. In case of less than 3 samples in at least one of the groups compared, qualitative assessment based on presence/absence of Cq values was performed; here ▲ and ▼ indicate significant ($p < 0.05$) upregulation and downregulations, respectively. Pleiotropic genes, marked with an asterisk, have been reallocated according to the results of Fig. 2B.

Clinical Sign			Innate								Th1-like				Th2-like						Th17-like					
Patogen of Interest	Oragan	Reference Pathogen	IL-1ß	IL-8	IL-6*	IL-10*	Cath. 1	Cath. 2	Lysozyme	SAA	IFNγ	IL-12	TGF ß*	TNF α	IL-4/13a	IgDm	IgDs	IgM	IgT	TCR ß	C3	IL-2	IL-17A/F2	IL17-C1	Il-17-C2	IL-22
Ichthyophthirius multifiliis	Gill	Aeromonas	↓	↓		↓	↑	↓	↓	↓	↑			↓	↑		↑	↑	↑	▼		↑			↓	↓
		Flavo				↓		↓	↓	↓	↓	↓	↓	↓	↑				↑	▼			↓	↓	↓	↓
		Vibrio		↓	↓		↑	↓	↓	↓	↑	↓	↓	↓	↑			↑	↑	▼			↓	↓	↓	↓
		Yersinia	↓	↓	↓	↓	↑	↓	↓	↓		↓	↓	↓	↑			↑	↑	▼		↓		↓	↓	↓
	Liver	Aeromonas	↓	↓	↓	↓	↓	↓	↓	↓	↓	↓	↓	↓	↓	↓						↓	↓	↓		↓
		Flavo			↓	↓	↓	↓		↓	↓	↓		↓		↓		↓		↓		↓		↓		↓
		Vibrio		↓	↓	↓	↓	↓		↓		↓		↓										▲		▲
		Yersinia	↓	↓	↓	↓	↓	↓	↓	↓	↓	↓	↓	↓	↓	↓		↓		↓		↓		↓		↓
	Spleen	Aeromonas	↓	↓	↓	↓	↓	↓		↓	↓	↓		↓			↓	↓	↓	↓		↓			↓	↓
		Flavo	↓	↓	↓	↓		↓		↓	↓	↓		↓		↑					↓	↓		↓		
		Vibrio	↓	↓	↓	↓	↓	↓	↑	↓	↓	↓		↓		↑	↑		↑			↓	↓			↓
		Yersinia	↓	↓	↓	↓		↓	↑	↓	↓	↓		↓		↑	↑	↑		↓	↓	↓	↓	↓	↓	↓
Aeromonas salmonicida	Gill	Flavo	↑	↑		↓		↓	↓	↓	↓				↓		↓			↓		↓		↓		↑
		Ich	↑	↑		↑	↓	↑	↑	↑	↓			↑	↓		↓	↓	↓	▲		↓			↑	↑
		Vibrio						↓		↓				↓						↓	↓	↓	↓	↓		
		Yersinia	↓		↓	↓	↓	↓		↓	↓	↓		↓	↓							↓		↓		
	Liver	Flavo	↑	↑	↑		↑		↑	↑			↑	↑	↑						↓	↑		↑	▼	↑
		Ich	↑	↑	↑	↑	↑	↑	↑	↑	↑	↑	↑	↑	↑	↑						↑	↑	↑		↑
		Vibrio		↓		↑	↑	↓	↑			↓			↑								▲	▲		▲
		Yersinia		↓			↑								↑		↑							↓	▼	
	Spleen	Flavo	↑	↑		↑	↑	↑		↑	↑	↑		↑	↑	↑	↑	↑	↑	↑		↑			↑	
		Ich	↑	↑	↑	↑	↑	↑		↑	↑	↑		↑			↑	↑	↑	↑		↑			↑	↑
		Vibrio		↓		↑		↑	↑			↓		↓	↑	↑	↑	↑	↑						↑	↓
		Yersinia		↓		↑	↑	↑	↑	↓		↓	↑	↑		↑	↑	↑	↑		↓		↓			
Flavobacterium psychrophilum	Gill	Aeromonas	↓	↓		↑		↑	↑	↑	↑				↑		↑			↑		↑		↑		↓
		Ich				↑		↑	↑	↑	↑	↑	↑	↑	↓				↓	▲			↑	↑	↑	↑
		Vibrio		↓		↑		↑			↑				↑	↑	↑				↓			↓		↓
		Yersinia		↓	↓	↑			↑			↓					↑					↓				↓
	Liver	Aeromonas	↓	↓	↓		↓		↓	↓			↓	↓	↓						↑	↓		↓	▲	↓
		Ich			↑	↑	↑	↑		↑	↑	↑		↑		↑		↑		↑		↑		↑		↑
		Vibrio		↓	↓	↑		↓		↓	↑	↓		↓		↑		↑					▲	▲	▲	▲
		Yersinia	↓	↓	↓				↓	↓	↓	↓	↓	↓	↓		↑	↑			↑	↓		↓	↓	↓
	Spleen	Aeromonas	↓	↓		↓	↓	↓		↓	↓	↓		↓	↓	↓	↓	↓	↓	↓		↓			↓	
		Ich	↑	↑	↑	↑		↑		↑	↑	↑		↑		↓					↑	↑		↑		
		Vibrio	↓	↓	↓	↓	↓		↑	↓	↓	↓		↓												↓
		Yersinia	↓	↓	↓				↑	↓	↓	↓		↓	↓					↓					↓	↓
Vibrio anguillarum	Gill	Aeromonas						↑		↑				↑						↑	↑	↑	↑	↑		
		Flavo		↑		↓		↓			↓				↓	↓	↓				↑			↑		↑
		Ich		↑	↑		↓	↑	↑	↑	↓	↑	↑	↑	↓			↓	↓	▲			↑	↑	↑	↑
		Yersinia				↓					↓				↓						↑		↑			
	Liver	Aeromonas						↑						↑								↓	▼	▼		▼
		Flavo		↑	↑	↓		↑		↑	↓	↑		↑		↓		↓					▼	▼	▼	▼
		Ich		↑	↑	↑	↑	↑		↑		↑		↑						↑				▼		▼
		Yersinia				↓		↑	↓		↓					↓				↓		↓	▼	▼	▼	▼
	Spleen	Aeromonas			↑			↓				↑			↓	↓	↓	↓						↑	↓	↑
		Flavo			↑				↓	↑	↑	↑		↑					↓		↓					↑
		Ich	↑		↑		↑	↑	↓	↑	↑	↑		↑		↓	↓		↓						↑	↑
		Yersinia				↑	↑			↓		↓		↑		↑	↑				↓					↑
Yersinia ruckeri	Gill	Aeromonas	↑		↑	↑	↑	↑		↑	↑	↑		↑	↑							↑		↑		
		Flavo		↑	↑	↓			↓			↑					↓					↑				↑
		Ich	↑	↑	↑	↑	↓	↑	↑	↑		↑	↑	↑	↓			↓	↓	▲		↑		↑	↑	↑
		Vibrio				↑					↑				↑						↓		↓			
	Liver	Aeromonas		↑			↓								↓		↓							↑	▲	
		Flavo	↑	↑	↑				↑	↑	↑	↑	↑	↑	↑		↓	↓			↓	↑		↑	↑	↑
		Ich	↑	↑	↑	↑	↑	↑	↑	↑	↑	↑	↑	↑	↑	↑		↑		↑		↑		↑	↑	↑
		Vibrio				↑		↓	↑		↑					↑				↑		↑	▲	▲	▲	▲
	Spleen	Aeromonas		↑		↓	↓	↓	↓	↑		↑	↓	↓		↓	↓	↓	↓		↑		↑			
		Flavo	↑	↑	↑				↓	↑	↑	↑		↑	↑					↑					↑	↑
		Ich	↑	↑	↑	↑		↑	↓	↑	↑	↑		↑		↓	↓	↓		↑	↑	↑	↑	↑	↑	↑
		Vibrio				↓	↓			↑		↑		↓		↓	↓				↑					↓
