# Supplementary material for: Differential immune gene expression in rainbow trout, Oncorhynchus mykiss (walbaum), exposed to five pathogens: Aeromonas salmonicida, Flavobacterium psychrophilum, Vibrio anguillarum, Yersinia ruckeri and Ichthyophthirius multifiliis
Source: Comp Immunol Rep. 2024 Sep 12;7:200166. doi: 10.1016/j.cirep.2024.200166 (PMC11437762; doi:10.1016/j.cirep.2024.200166)
Supplement: Supplementary file 11 [file mmc11.pdf]

S10. No clinical signs. gene expression analysis comparing the pathogens to each other's. Significant quantitative up- and downregulations (fold at least 2 and $p < 0.5$ of one-way ANOVA) relative to reference pathogens are indicated by ↑ and ↓, respectively. In case of less than 3 samples in at least one of the groups compared, qualitative assessment based on presence/absence of Cq values was performed; here ▲ and ▼ indicate significant ($p < 0.05$) upregulation and downregulations, respectively. Pleiotropic genes, marked with an asterisk, have been reallocated according to the results of Fig. 2B.

**No Clinical Sign**			**Innate**								**Th1-like**				**Th2-like**						**Th17-like**					
Patogen of Interest	Oragan	Reference Pathogen	IL-1ß	IL-8	IL-6*	IL-10*	Cath. 1	Cath. 2	Lysozyme	SAA	IFNγ	IL-12	TGF ß*	TNF α	IL-4/13a	IgDm	IgDs	IgM	IgT	TCR ß	C3	IL-2	IL-17A/F2	Il17-C1	Il-17-C2	IL-22
Ichthyophthirius multifiliis	Gill	Aeromonas			▲	↑	↑				↑	↑			↑			↑	↑	▼					▲	
		Flavo		↑	↑		↑	↑		↑		↑			↑	↓		↑		▼				↓	↓	
		Vibrio			▲		↑													▼	↓		↓	↓		
		Yersinia		↑			↑		↓						↑			↑	↑	▼					↓	
	Liver	Aeromonas	↓		↓	↓	↓	↓		↓	↓	↓	↓	↓	↓		↓	↓	↓	↓		↓		↓		↓
		Flavo	↓	↑		↓	↑		↑	↑	↓	↓		↓		↓	↓		↓	↓		↓		↓		↓
		Vibrio	↓	↓	▲	↓	↓	↓	↑	↓	↓	↓		↓			↓		↓	↓		↓		▲		▲
		Yersinia	↓		↓	↓	↓	↓		↓	↓			↓		↓	↓		↓	↓		↓		↓		↓
	Spleen	Aeromonas	↓	↓		↓		↓		↓	↓			↓		↓	↓		↓			↓		↓	↓	
		Flavo				↑	↑	↑	↑	↑	↓				↑	↓	↓		↓	↓				↓		
		Vibrio	↓	↓	↓				↑	↓	↓	↓		↓												↓
		Yersinia	↓	↓	↓	↓	↓	↓		↓	↓	↓		↓				↑				↓			↓	
Aeromonas salmonicida	Gill	Flavo	↑					↑		↑						↓	↓			↓	↓			↓	↓	
		Ich				↓	↓				↓	↓			↓			↓	↓	▲					↑	
		Vibrio				↓															↓		▲			
		Yersinia														↓										
	Liver	Flavo	↑	↑	↑	↑	↑	↑	↑	↑	↑		↑	↑	↑					↑	↑	↑	↑	↑	▼	↑
		Ich	↑		↑	↑	↑	↑		↑	↑	↑	↑	↑	↑		↑	↑	↑	↑		↑		↑		↑
		Vibrio		↓	▲	↑			↑						↑									▲	▼	▲
		Yersinia								↓					↑		↑								▼	↑
	Spleen	Flavo	↑	↑		↑	↑	↑	↑	↑	↑				↑			↑		↓		↑	↓		↑	
		Ich	↑	↑		↑		↑		↑	↑			↑		↑	↑		↑			↑		↑	↑	
		Vibrio							↑								↑	↑	↑							
		Yersinia					↓			↓		↓				↑	↑	↑	↑							
Flavobacterium psychrophilum	Gill	Aeromonas	↓		▲			↓		↓						↑	↑			↑	↑			↑	▲	
		Ich		↓	↓		↓	↓		↓		↓			↓	↑		↓		▲				↑	↑	
		Vibrio			▲					↓																
		Yersinia			↓			↓		↓										↑	↑			↑	↑	↑
	Liver	Aeromonas	↓	↓	↓		↓	↓	↓	↓	↓		↓	↓	↓					↓	↓	↓	↓	↓	▲	↓
		Ich	↑	↓		↓	↓		↓	↓	↑	↑		↑		↑	↑		↑	↑		↑		↑		↑
		Vibrio		↓	↑		↓	↓		↓				↓								↓		▲		▲
		Yersinia	↓	↓		↑	↓	↓	↓	↓	↓		↓	↓			↑					↓			↓	
	Spleen	Aeromonas	↓	↓		↓	↓	↓	↓	↓	↓				↓			↓		↑		↓	↑		↓	
		Ich				↓	↓	↓	↓	↓	↑				↓	↑	↑		↑	↑				↑		
		Vibrio		↓	↓	↓	↓	↓		↓	↓	↓		↓	↓	↑	↑			↑		↓				
		Yersinia	↓	↓	↓	↓	↓	↓	↓	↓		↓		↓	↓	↑	↑	↑		↑	↓	↓			↓	
Vibrio anguillarum	Gill	Aeromonas				↑															↑				↑	
		Flavo			↑					↑																
		Ich					↓													▲	↑		↑	↑	↑	
		Yersinia																			↑					
	Liver	Aeromonas			↑	↓															↓		▼	▼		▼
		Flavo		↑	↑		↑	↑		↑				↑								↑		▼		▼
		Ich	↑	↑	↑	↑	↑	↑	↓	↑	↑	↑		↑			↑		↑	↑		↑		▼		▼
		Yersinia			↑	↓			↓			↑				↓							▼	▼		▼
	Spleen	Aeromonas						↓								↓	↓	↓								
		Flavo	↑		↑		↑			↑	↑	↑			↑	↓			↓	↓	↑	↑				↑
		Ich						↑	↓	↑	↑	↑		↑											↑	↑
		Yersinia							↓															↑	↓	
Yersinia ruckeri	Gill	Aeromonas				↑										↑										
		Flavo			↑			↑		↑										↓	↓			↓	↓	↓
		Ich		↓			↓		↑						↓			↓	↓	▲					↑	
		Vibrio																			↓					
	Liver	Aeromonas								↑					↓		↓								▲	↓
		Flavo	↑	↑		↑	↑	↑	↑	↑	↑		↑	↑			↓					↑			↑	
		Ich	↑		↑	↑	↑	↑		↑	↑			↑		↑	↑		↑	↑		↑		↑		↑
		Vibrio			▲	↑			↑			↓				↑								▲		▲
	Spleen	Aeromonas					↑			↑		↑				↓	↓	↓	↓							
		Flavo	↑	↑	↑	↑	↑	↑	↑	↑		↑		↑	↑	↓	↓	↓		↓	↑	↑			↑	
		Ich	↑	↑	↑	↑	↑	↑		↑	↑	↑		↑				↓				↑			↑	
		Vibrio							↑															↓	↑	
