# Supplementary material for: Differential immune gene expression in rainbow trout, Oncorhynchus mykiss (walbaum), exposed to five pathogens: Aeromonas salmonicida, Flavobacterium psychrophilum, Vibrio anguillarum, Yersinia ruckeri and Ichthyophthirius multifiliis
Source: Comp Immunol Rep. 2024 Sep 12;7:200166. doi: 10.1016/j.cirep.2024.200166 (PMC11437762; doi:10.1016/j.cirep.2024.200166)
Supplement: Supplementary file 12 [file mmc12.pdf]

S11. Survivors. Gene expression analysis comparing the pathogens to each other's. Significant quantitative up- and downregulations (fold at least 2 and $p < 0.5$ of one-way ANOVA) relative to reference pathogens are indicated by ↑ and ↓, respectively. In case of less than 3 samples in at least one of the groups compared, qualitative assessment based on presence/absence of Cq values was performed; here ▲ and ▼ indicate significant ($p < 0.05$) upregulation and downregulations, respectively. Pleiotropic genes, marked with an asterisk, have been reallocated according to the results of Fig. 2b.

Survivors			Innate								Th1-like				Th2-like						Th17-like					
POI	Oragan	Reference Pathogen	IL-1ß	IL-8	IL-6*	IL-10*	[illegible]	[illegible]	Lysozyme	SAA	IFNγ	IL-12	TGF ß*	TNF α	IL-4/13a	IgDm	IgDs	IgM	IgT	TCR ß	C3	IL-2	IL-17A/F2	IL17-C1	Il-17-C2	IL-22
Ichthyophthirius multifiliis	Gill	Aeromonas	↑	↑	▲	↑	↑	↑		↑	↑	↑		↑			↑	↑				↓	↓		▲	
		Flavo	↑		↑	↑	↑	↑		↑	↑	↑		↑			↑				↓	↓	↓	↓	▲	↓
		Vibrio	↑		▲	↑	↑	↑		↑					↓	↓	↑	↑				↓			↓	
		Yersinia	↑	↑	↑	↑	↑	↑		↑	↑	↑		↑			↑	↑		↑		↓			↓	
	Liver	Aeromonas	↓	↑				↑	↑	↑	↓		↓	↓		↓		↓				↓				
		Flavo	↓	↑	↓			↑	↑	↑	↓		↓	↓	↓	↓	↑				↑	↓		↓		↓
		Vibrio		↑	▲		↑	↑	↑						↓	↓	↑				↑	↓				
		Yersinia	↓	↑	↓				↑	↑	↓	↓	↓	↓	↓	↓		↓			↑	↓		↓		
	Spleen	Aeromonas							↑	↑			↓			↓		↓	↓	↓	↑					
		Flavo				↑		↑	↑	↑	↓		↓	↑		↓				↓	↑					
		Vibrio	↑				↑	↑	↑	↑	↓		↓	↑		↓		↓	↓	↓	↑	↓	↓			
		Yersinia	↓	↓					↑	↑	↓		↓			↓	↑			↓	↑			↓		
Aeromonas salmonicida	Gill	Flavo			↓			↑				↓	↓								↓			↓	↓	
		Ich	↓	↓	↓	↓	↓	↓		↓	↓	↓		↓			↓	↓				↑	↑			
		Vibrio		↓	↓						↓		↓		↓	↓								↓		
		Yersinia			↓	↑				↓													↑			↑
	Liver	Flavo		↑	▼				↑	↑											↑				▼	
		Ich	↑	↓	▼			↓	↓	↓	↑		↑	↑		↑		↑				↑			▼	
		Vibrio		↑	▲									↑							↑					
		Yersinia					↓							↑	↓								↑			
	Spleen	Flavo				↑	↑	↑	↑	↑							↑	↑			↑					
		Ich							↓	↓			↑			↑		↑	↑	↑	↓					
		Vibrio					↑	↑		↑							↑									
		Yersinia															↑	↑		↓				↓		
Flavobacterium psychrophilum	Gill	Aeromonas			▲			↓				↑	↑								↑			↑	▲	
		Ich	↓		↓	↓	↓	↓		↓	↓	↓		↓			↓				↑	↑	↑	↑	▲	↑
		Vibrio			▲			↓						↓	↓									↑		↑
		Yersinia		↑		↑		↓		↓	↑	↑									↑		↑	↑	↑	↑
	Liver	Aeromonas		↓					↓	↓											↓					
		Ich	↑	↓	↑			↓	↓	↓	↑		↑	↑	↑	↑	↓				↓	↑	▲	↑		↑
		Vibrio			↑					↓				↑												↑
		Yersinia							↓	↓																
	Spleen	Aeromonas				↓	↓	↓	↓	↓							↓	↓			↓					
		Ich				↓		↓	↓	↓	↑		↑	↓		↑				↑	↓					
		Vibrio				↓		↓	↓	↓									↓							
		Yersinia	↓			↓				↓	↓			↓						↓	↓					
Vibrio anguillarum	Gill	Aeromonas		↑	↑						↑		↑		↑	↑								↑		
		Flavo						↑						↑	↑									↓		↓
		Ich	↓		↓	↓	↓	↓		↓					↑	↑	↓	↓				↑			↑	
		Yersinia		↑							↑					↑	↑									
	Liver	Aeromonas	↓	↓	▼								↓											↓		
		Flavo			▼					↑				↓									▼		▼	↓
		Ich		↓	▼		↓	↓	↓						↑	↑	↓				↓	↑			▼	
		Yersinia			▼		↓						↓	↓	↓						↓			↓		
	Spleen	Aeromonas	↓				↓			↓											↓					
		Flavo	↓			↑		↑	↑	↑																
		Ich	↓				↓	↓	↓	↓			↑					↑	↑		↓	↑				
		Yersinia									↓		↓				↑									
Yersinia ruckeri	Gill	Aeromonas			↑	↓				↑													↓			↓
		Flavo		↓		↓		↑		↑	↓	↓									↓		↓	↓	↓	↓
		Ich	↓	↓	↓	↓	↓	↓		↓	↓	↓		↓			↓	↓		↓		↑			↑	
		Vibrio		↓							↓					↓	↓									
	Liver	Aeromonas					↑							↓	↑								↓			
		Flavo							↑	↑																
		Ich	↑	↓	↑				↓	↓	↑	↑	↑	↑	↑	↑		↑			↓	↑		↑		
		Vibrio			▲		↑						↑	↑	↑						↑			↑		
	Spleen	Aeromonas															↓	↓		↑				↑		
		Flavo	↑			↑				↑	↑			↑						↑	↑					
		Ich	↑	↑					↓	↓	↑		↑			↑	↓			↑	↓			↑		
		Vibrio									↑		↑				↓									
